# Growth in biofilms prepares *Mycobacterium avium* subsp. *hominissuis* for the macrophage microenvironment

**DOI:** 10.3389/fmicb.2025.1709239

**Published:** 2025-12-04

**Authors:** William R. McManus, Katie Mulvey, Elizabeth M. Brooks, Sheri A. Sanders, Jeffrey S. Schorey

**Affiliations:** Department of Biological Sciences, Galvin Life Science Center, University of Notre Dame, Notre Dame, IN, United States

**Keywords:** macrophages, RNAseq, biofilm, *Mycobacterium avium*, DGE

## Abstract

**Introduction:**

*Mycobacterium avium* subs. *hominissuis* is an opportunistic pathogen, causing pulmonary infections in individuals who are immunocompromised or whose respiratory systems are damaged due to injuries or diseases such as cystic fibrosis, bronchiectasis, or chronic obstructive pulmonary disease. *M. avium* is an environmental microbe commonly found in numerous natural and engineered habitats, including marginal niches where few species are able to survive. *M. avium* is able to persist in these habitats through biofilm formation, a process in which the hydrophobic mycobacterial cells preferentially adhere to a surface or to particles in suspension. The process of biofilm formation as well as the movement of *M. avium* from its environmental niche into a host entails a large shift in conditions and stressors. To mediate these transitions, it is necessary that *M. avium* responds by modulating its gene expression.

**Methods:**

We used next generation sequencing to define changes in *M. avium* gene expression that occur during biofilm formation and during transition to survival inside host macrophages.

**Results:**

We identified genes that are differentially expressed in two different *M. avium* biofilm models relative to planktonic *M. avium*. We also identified genes that show differential expression in *M. avium* isolated from both infected macrophages and in biofilms when compared to planktonic *M. avium*. For some of these genes, we did not observe notable changes in expression in biofilm-grown *M. avium* pre- and post-infection, suggesting that genes expressed during residence in a biofilm may condition *M. avium* to survive in a macrophage.

**Discussion:**

Overall, these results identify a number of genes that may be important for biofilm formation and survival within macrophages and which will provide new foci for drug development that targets biofilm formation and *M. avium* virulence.

## Introduction

*Mycobacterium avium* subsp. *hominissuis* is an opportunistic pathogen that causes pulmonary infections in people whose respiratory systems have been damaged due to injuries; in individuals with respiratory diseases such as cystic fibrosis, bronchiectasis, or chronic obstructive pulmonary disease; or, increasingly, in elderly people without predisposing conditions, especially in elderly women with slender physiques (Kwon et al., 2019). Disseminated infections are seen in people who are immunocompromised (Kwon et al., 2019). MAH is present in various environmental sources, including showerheads and hot tubs, and evidence supports these environmental reservoirs as major sources of MAH for human infections (Falkinham, 2009; Feazel et al., 2009). Infections with MAH occur worldwide, and the incidence of these infections is increasing (Dahl et al., 2022; Prevots et al., 2023).

The transition of MAH from an environmental niche to a host entails a large shift in the conditions and stressors experienced by mycobacteria. In the typical route of infection, MAH detaches from an environmental biofilm, is aerosolized, and then inhaled by a potential human host. To establish an infection, MAH must first survive on the respiratory epithelium and then survive intracellularly after being engulfed by alveolar macrophages. For MAH to survive this transition, it is necessary that the bacterium responds to its change in habitat by modulating the expression of genes involved in various processes (Yamazaki et al., 2006a,b). It is also possible that the patterns of gene expression developed during residence in a biofilm may condition MAH for survival in the host. This hypothesis is based on observations of increased drug resistance of biofilm MAH and the recalcitrance of MAH infections to therapy (McNabe et al., 2011; Chakraborty et al., 2021; Chakraborty and Kumar, 2019), phenotypes that could have a similar genetic basis. Studies have suggested that the resistance phenotype in biofilm-grown MAH is primarily based on gene expression, as opposed to other factors. Heightened resistance of biofilm-grown MAH to chlorine and the antibiotics clarithromycin and rifamycin is preserved when the bacteria are resuspended, indicating that individual bacterial phenotypes, more so than a physical barrier imposed by the biofilm ECM, lead to resistance (Steed and Falkinham, 2006; Falkinham, 2007). Furthermore, the susceptibility of the resuspended biofilm cells to chlorine after 1 day of growth in suspension decreases to normal levels for planktonic cells, indicating that the heightened resistance is due, in part, to adaptations in gene expression rather than mutation-driven selection of genetically resistant subpopulations (Steed and Falkinham, 2006).

Previous studies that have characterized genes transcribed by *M. avium* under various conditions have done so with the use of nucleic acid microarrays, promoter library screens, and/or qRT-PCR (Hou et al., 2002; Danelishvili et al., 2004; Motamedi et al., 2014). Although these tools have yielded important information about gene expression in *M. avium*, they are limited by a number of factors, including the inability to detect unknown transcripts. More recently, next-generation sequencing (NGS) technologies have enabled a more unbiased, non-targeted characterization of all genes expressed in a biological sample using direct sequencing of transcripts in cDNA libraries and detection of expression over a greater dynamic range than microarrays (Wang et al., 2009). While these technologies have been frequently used to study *M. tuberculosis* (Kundu and Basu, 2021; Bhattacharjee et al., 2022) and several species of rapidly growing mycobacteria (Dubois et al., 2019; Li et al., 2017; Liu et al., 2016; Liu et al., 2016), their application to *M. avium* has been limited. Ignatov et al. (2010) used the early NGS technology, 454 sequencing, to compare the *M. avium* transcriptome in bacteria used to infect a susceptible mouse strain and a resistant mouse strain 13 weeks after respiratory infection (Ignatov et al., 2010). To our knowledge, no studies have examined global gene expression under different biofilm conditions and how this compares to gene expression under planktonic and infection conditions.

In this study, we used Illumina RNA sequencing (RNA-seq) to characterize differences in global gene expression in MAH across multiple biologically relevant conditions. These conditions include residence in two biofilm models, as well as growth in liquid culture. In the first biofilm model, cells of MAH were incubated in a nutrient-poor medium for 4 weeks (Freeman et al., 2006; Recht and Kolter, 2001; Van Wyk et al., 2017), allowing biofilm formation over an extended period that may reflect the gradual process of biofilm formation in the environment (Falkinham, 2018). The second biofilm model used dithiothreitol (DTT)-induced thiol reductive stress to trigger rapid biofilm development, with biofilm formation occurring in one day (Trivedi et al., 2016; Chakraborty et al., 2021; Mavi et al., 2023), which may represent a biofilm formation response that could occur *in vivo*, where MAH could experience intracellular thiol reductive stress from a host factor like glutathione (Mavi et al., 2023; Morris et al., 2013). MAH from liquid culture or from biofilms generated in nutrient-poor media were also used to infect RAW264.7 macrophages for 24 h to examine transcriptional responses to infection and to define genes expressed in biofilms that may promote adaptation to the macrophage environment.

## Materials and methods

### Bacterial strains and culture conditions

*Mycobacterium avium* subsp. *hominissuis* strain A5 was originally isolated from the blood of an AIDS patient with a disseminated mycobacterial infection. MAH was cultured on Middlebrook 7H10 agar (BD Biosciences, NJ, USA) containing 10% oleic acid albumin dextrose catalase (OADC; BD Biosciences) supplement at 37 °C and in Middlebrook 7H9 broth (BD Biosciences) containing 10% OADC at 37 °C with constant agitation. For biofilms developed in nutrient-poor media, MAH was grown in liquid culture to an optical density at 600 nm (OD600) of approximately 0.8, then pelleted and resuspended in biofilm media to an OD600 = 0.2. The biofilm media consisted of M63 media (United States Biological, MA, USA) supplemented with 2% glucose, 0.5% Casamino Acids, 1 mM MgSO_4_, and 0.7 mM CaCl_2_ (Freeman et al., 2006; Recht and Kolter, 2001). A volume of 1 mL of the suspension was dispensed into each well of multiple 24-well polystyrene plates, and the plates were sealed with parafilm and incubated at 37 °C for 4 weeks. PBS was added to the inter-well spaces to prevent evaporation. For biofilm formation using dithiothreitol (DTT), MAH was grown in liquid culture to an OD600 of approximately 0.8 and then pelleted and resuspended in 7H9 broth + 5% OADC to an OD600 = 0.8. DTT (Roche, Basel, Switzerland) was added to a final concentration of 6 mM (Trivedi et al., 2016; Chakraborty et al., 2021). A volume of 1 mL of the suspension was dispensed into each well of multiple 24-well polystyrene plates, and the plates were incubated at 37 °C for 24 h.

### RAW264.7 culture and infection

RAW264.7 macrophages (ATCC# TIB-71) were cultured in Dulbecco’s Modified Eagle Medium (DMEM) supplemented with 10% fetal bovine serum (FBS) at 37 °C and 5% CO_2_. Penicillin (100 U/mL) and streptomycin (100 μg/mL) were included in the culture media, unless otherwise noted. For infection with mycobacteria from liquid cultures or biofilms, macrophages were seeded onto two T175 tissue culture flasks in antibiotic-free medium and grown to confluence. To prepare biofilm samples for infection, biofilm media supernatants were removed, and the biofilms were rinsed with tissue culture-grade PBS (TC-PBS). The biofilms were then resuspended in TC-PBS, and multiple wells were pooled on ice. Biofilm associations were disrupted by three 10 s sonication bursts, each followed by a 60 s recovery. Bacteria in liquid culture and disrupted biofilm suspensions were quantified using an Apogee Micro flow cytometer (Apogee Flow Systems, Hertfordshire, UK). Prior to infection, bacteria from the liquid culture and biofilms were opsonized at 37 °C for 2 h in DMEM+10% normal human serum+0.5% BSA. The bacteria were then pelleted out of the opsonizing media by centrifugation at 13,000 rpm for 90 s and resuspended in DMEM+10% FBS. Appropriate volumes of the bacterial cell suspension were then added to 20 mL antibiotic-free DMEM+10% FBS on each RAW264.7 culture to achieve a multiplicity of infection of approximately 3:1, confirmed by quantification of CFU in the cell lysate post-infection. After 4 h of inoculation, the infection supernatants were removed, and the monolayers were rinsed with TC-PBS. Fresh antibiotic-free DMEM+10% FBS was then added, and the cultures were incubated under normal conditions until 24 h post-infection. At the infection endpoint, the media supernatant was removed or discarded. Monolayers were resuspended by scraping in TC PBS, and then the resuspended cells were pelleted by centrifugation for 7.5 min at 3,500 rpm. The RAW264.7 cells were then selectively lysed by resuspension in water containing 0.02% sodium dodecyl sulfate (SDS). The suspension was pelleted and rinsed with lysis buffer, pelleted, and rinsed with water to reduce residual SDS, and then the liberated bacteria were pelleted and resuspended in Buffer RLT containing 1% *β*-mercaptoethanol (Acros Organics, Geel, Belgium) for total RNA extraction.

### Total RNA extraction

Bacterial samples isolated from liquid culture, biofilms, or infection conditions were pelleted, then resuspended in 300 μL Buffer RLT (QIAGEN, Hilden, Germany) containing 1% β-mercaptoethanol (Acros Organics). Samples were then transferred to bead-beating tubes containing 100 μL silica disruption beads and then stored at −80 °C. For lysis, samples were thawed on ice and then disrupted in a Mini-BeadBeater-16 (Biospec Products, Oklahoma, United States) for three rounds of 30 s each, with a 60-s recovery on ice following each round of bead beating. Beads and debris were pelleted by centrifugation at 13,000 rpm for 5 min at 4 °C. The lysates were isolated from the pelleted material, combined with 300 μL 70% ethanol, and RNA was purified following the Qiagen RNeasy Mini Kit protocol, including on-column DNase treatment (QIAGEN). The efficacy of the on-column DNase treatment was verified by PCR. Quality and quantity of RNA were assessed using a Nanodrop (Thermo Scientific, MA, USA) and Bioanalyzer (Agilent, CA, USA). Total RNA was eluted in water and stored at −80 °C.

### RNA sequencing

Total RNA was assessed for quality using the Agilent Tapestation and RNA Screentape for prokaryotic analysis (Agilent Technologies, CA, USA). A total of 5 replicate MAH isolates were processed for RNA-seq for planktonic, DTT biofilm, and planktonic/infection samples, and 4 replicates were processed for M63 biofilm and M63 biofilm/infection samples. RNA libraries were prepared at the University of Notre Dame Genomics & Bioinformatics Core Facility using the NEBNext rRNA depletion kit (bacteria) employing the NEBNext RNase H-based RNA depletion workflow to target the removal of rRNA (New England Biolabs, MA, USA). The libraries were assessed for quality using KAPA Hi-Fi qPCR (Roche, Basel, Switzerland), the Agilent Tapestation DNA HS 1000 chip, and Qubit HS DNA (Thermo Fisher Scientific, MA, USA) assays. MAH samples isolated from macrophages (Feazel et al., 2009) were weighted in the final library pool to achieve 320 million raw reads for each sample on average, whereas the samples from planktonic or biofilm cultures (13) yielded 41 million raw reads per sample on average. Sequencing was performed at the Indiana University School of Medicine Center for Medical Genomics on one lane of a NovaSeq 6,000 S4 (Illumina, CA, USA) 200-cycle flow cell using paired 100 bp reads.

### Bioinformatics

Raw sequences were processed using Trimmomatic (Bolger et al., 2014) and Sickle (Joshi and Fass, 2011) to remove the adaptor sequences. rRNA and contaminating host sequences were removed using BBMap (Bushnell, 2014). Quality of sequences was evaluated using FastQC before and after the processing steps. Cleaned sequences were aligned to the MAH reference genome from the NCBI Genome database (GenBank GCA_000696715.1 https://www.ncbi.nlm.nih.gov/datasets/genome/?taxon=1160711) using HISAT2 (Kim et al., 2019). Read counts per genome feature (using the NCBI GenBank annotation) were quantified using HTSeq (Anders et al., 2015). Library normalization was carried out, and differential expression between conditions was analyzed using the freeCount (v0.1) differential analysis R Shiny application built on the edgeR package (Brooks et al., 2024). Thresholds for significant differential expression in the edgeR pairwise tests were set at log_2_ fold change of >2 or <−2 and a false discovery rate (FDR) of <0.01. We used these FDR and LFC cutoffs to identify genes that were both significantly and highly differentially expressed. The LFC threshold was set to limit the analysis of genes with high levels of DE between the experimental groups. Venn diagram comparisons of differentially expressed genes under different conditions were created using a shiny application of the ggVennDiagram package in R (Brooks, 2023; Gao et al., 2024). For gene ontology (GO) analysis, the proteome annotation tool PANNZER2 was used to assign GO classifications to the protein sequences corresponding to the MAH reference genome (Törönen and Holm, 2022). Significant enrichment of GO terms under different conditions was determined using the freeCount FA and topGO packages to perform Fisher’s exact tests in R. GO enrichment revealed gene sets and pathways with more differentially expressed genes than expected, given the background set of all other genes that were expressed in the experiment (Brooks, 2023). Similarities between MAH genes of interest and orthologs in *M. tuberculosis* were determined using NCBI BLAST.

### Quantitative PCR

To confirm the RNA-seq on a subset of genes, RNA was isolated from MAH grown under planktonic or biofilm conditions or from infected macrophages, as described above. For the three genes analyzed, nucleic acid sequences were collected from the GenBank MAH reference genome. Forward and reverse primers targeting each gene were designed using NCBI’s Primer-BLAST software, and each primer pair was validated via PCR and gel electrophoresis preceding qPCR. The primer pairs used are listed below:

RS22425: F: AGCTGGTGGAGTTGACGATC, R: ACCAACTTCTTCGGCGTCAA;
RS22460: F: ACTGCTCGAAATCCTCCAGC, R: ATCGACAACCCGTTCAACGA;
TetR: F: CCGATCAATGTCCTCGTCGT, R: CTCATGTCGGGCAGCATCA;
16S rRNA housekeeping gene:
F: CACTGGGACTGAGATACGGC, R: CCACCTACCGTCAATCCGAG.

To prepare cDNA for qPCR, 500 ng of RNA from each condition was used to synthesize cDNA using the SuperScript III reverse transcriptase kit (Invitrogen, Thermo Fisher Scientific, MA, USA). qPCR master mix (PowerUp™ SYBR™ Green Master Mix, Thermo Fisher Scientific, MA, USA) was prepared per manufacturer’s instructions with 1 μL of 10 μM primer working stocks. Mastermix was distributed to each well of a 96-well plate, and 10 ng of cDNA from each condition was added to three replicate wells. Plates were sealed with RT optical film (USA Scientific, FL, USA), and qPCR was conducted using the Applied Biosystems QuantStudio5 thermocycler (Thermo Fisher Scientific). The CT values of each target gene were normalized to the 16 s housekeeping gene, and a 2^-(∆∆CT) fold change was used to calculate the expression of each target gene.

## Results

### MAH RNA sample characteristics

**Figure 1:**
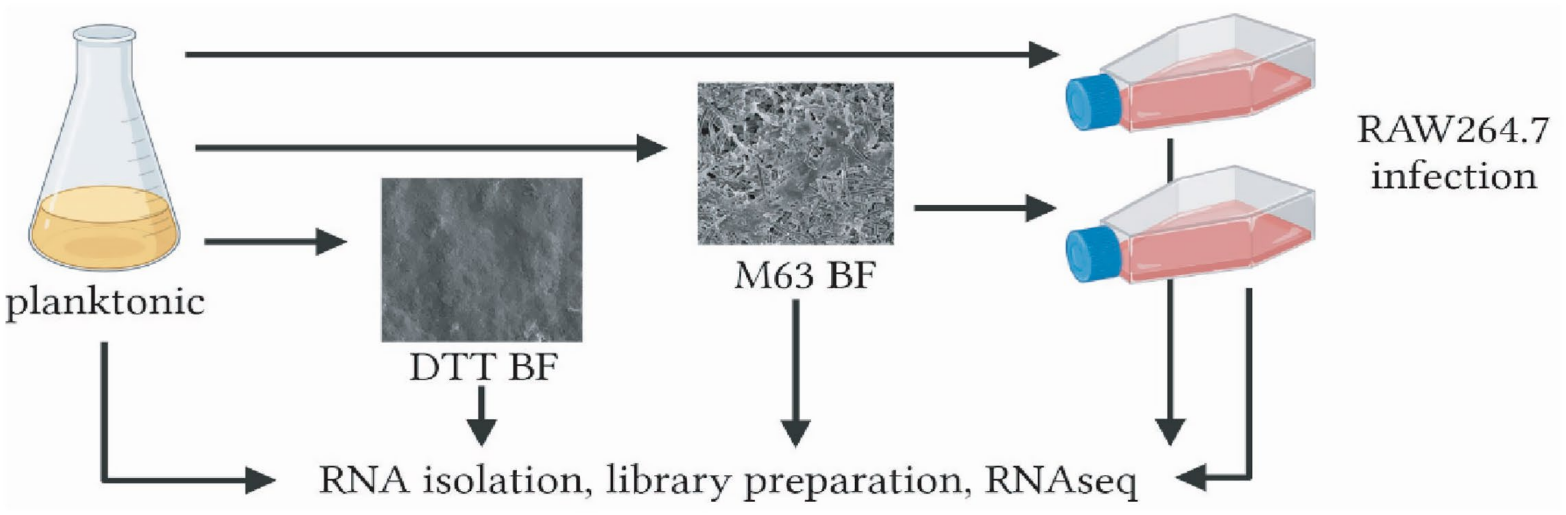
Schematic representation of *M. avium* samples generated for RNA-seq. MAH strain A5 was grown in planktonic culture in 7H9 broth+10% OADC and plated under different biofilm culture conditions. Bacteria from planktonic and M63 biofilm cultures were used to infect RAW264.7 macrophages for 24 h. Samples from both biofilms, planktonic culture, and macrophage infections were stored at −80 °C prior to RNA extraction and sequencing. BF, biofilm; DTT, dithiothreitol; OADC, oleic albumin dextrose catalase.

**Figure 2:**
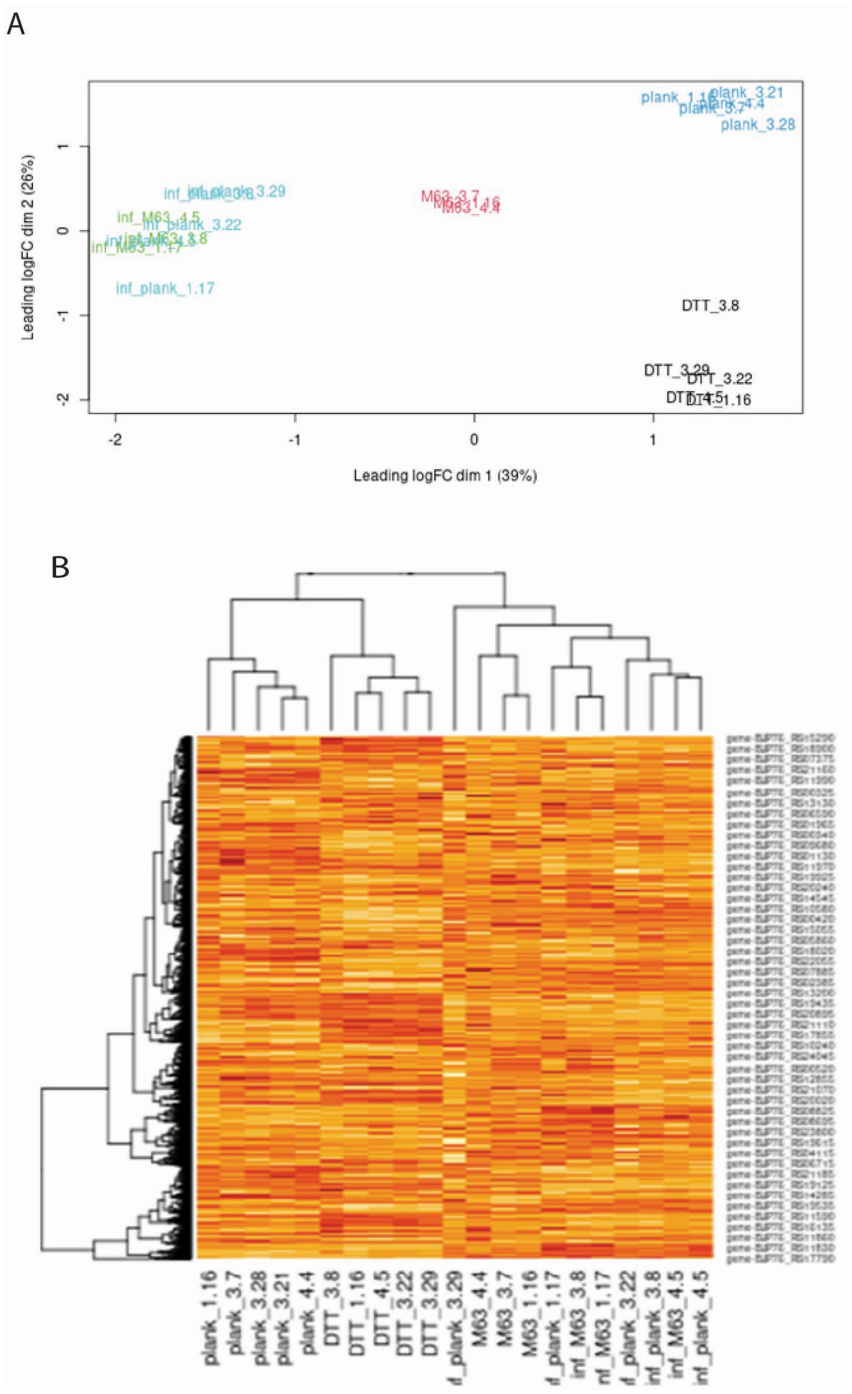
Sample clustering characteristics based on differences in gene expression. MDS plot **(A)** and heatmap **(B)** of all genes in all samples following library normalization using edgeR. Labels correspond to the following conditions: plank, planktonic culture-grown MAH; DTT, bacteria grown in DTT-induced biofilms; M63, bacteria grown in M63-incubated biofilms; inf_plank, planktonic bacteria isolated after 24-h infection of RAW264.7 macrophages; inf_M63, M63 biofilm-grown bacteria isolated after 24-h infection of RAW264.7 macrophages. For the heatmap, hierarchical clustering of individual samples is based on the calculated log2 CPM expression values. CPM, counts per million; DTT, dithiothreitol; MDS, multidimensional scaling.

We characterized patterns of gene expression in the MAH strain A5 when bacteria were grown in planktonic liquid culture and under biofilm conditions. We used biofilms formed over 4 weeks when grown in a nutrient-poor M63 medium plus supplement (M63 biofilms) and biofilms formed over 24 h when MAH is exposed to DTT (DTT biofilms). We also isolated mycobacteria from Raw264.7 cells infected for 24 h with either planktonic or M63 biofilm-grown bacteria (Figure 1). Mycobacterial RNA was isolated under each condition and subjected to RNA-seq. Genes were identified using HISAT2 to map reads to features in the GenBank annotation of the MAH reference genome and HTSeq to quantify the number of reads per feature. After cleaning the sequences and removing rRNA and contaminating host RNA, the libraries were normalized, and the differential expression of genes was calculated. Following normalization, 4,281 genes (of the 4,581 protein-coding genes annotated) had sufficient coverage for downstream expression analyses. The multidimensional scaling (MDS) plot and heatmap clustering revealed that the planktonic and biofilm culture samples clustered with their respective biological replicates (Figure 2). The DDT cluster was orthogonal to the trajectory of the M64 and infection cultures, suggesting that there is a different mechanism for rapid versus slow biofilm formation. The M63 biofilm and planktonic MAH samples were isolated from RAW264.7 macrophage cells after a 24-h infection clustered together, irrespective of the bacterial growth condition prior to the infection, suggesting that the environment in the macrophage causes similar transcriptional responses in the bacteria and minimizes the expression differences between planktonic and biofilm MAH observed prior to infection.

### Differential gene expression in MAH biofilms

**Figure 3:**
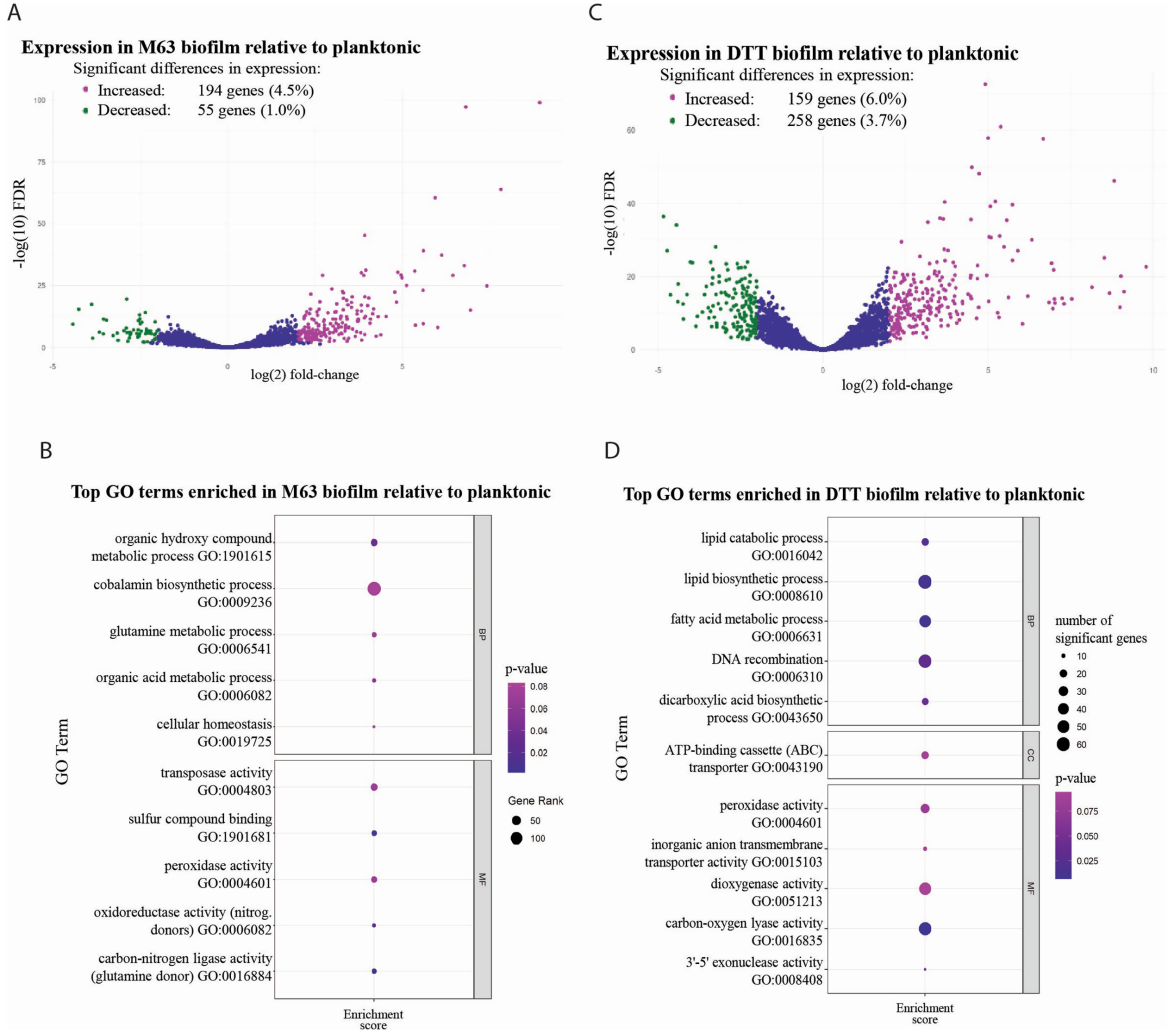
Differential gene expression and GO term enrichment in *M. avium* M63 and DTT biofilms relative to bacteria in planktonic culture. Volcano plots showing log_2_ fold expression changes of individual genes in the MAH M63 **(A)** and DTT **(C)** *in vitro* biofilm model. Significant changes in gene expression, indicated by green or purple coloring, were defined as having a fold-change (log_2_) > 2 and FDR < 0.01 using Volcano plots were created using edgeR. Dot plots illustrating the top enriched GO terms in MAH in the M63 **(B)** and DTT **(D)** biofilm models, relative to planktonic MAH. The threshold for inclusion was set at a weighted Fisher’s exact *p*-value of <0.1. Up to five terms are displayed per category. Dot plots were created using topGO in R. DTT, dithiothreitol; FDR, false discovery rate; GO, gene ontology.

Differential gene expression (DGE) analysis was used to characterize changes in gene expression accompanying the transition of MAH from a planktonic state, in laboratory planktonic culture, to a biofilm state, using either the M63-incubated or DTT-induced models for biofilm formation. The thresholds for defining differential expression were a fold-change in expression of >2 or <−2 and a false discovery rate (FDR) of <0.01. In MAH biofilms grown by a four-week incubation in M63 media, 249 genes (5.8% of the total set of genes analyzed) were expressed differentially, with 194 genes (4.5%) exhibiting increased expression in the biofilm and 55 genes (1.0%) exhibiting decreased expression (Figure 3A; Supplementary Table S1). GO analysis of genes with predicted involvement in biological processes revealed that MAH grown in M63 biofilms had enriched expression of genes involved in cellular homeostasis and multiple metabolic processes, including organic acid metabolism, glutamine metabolism, cobalamin biosynthesis, and organic hydroxy compound metabolism. Analysis of gene product molecular function GO annotations indicated significant enrichment of genes encoding proteins with functions including sulfur compound binding, catalysis of oxidation–reduction reactions with nitrogenous compounds as electron donors, catalysis of carbon-nitrogen ligation reactions where glutamine is the amido-N-donor, transposase activity, and peroxidase activity (Figure 3B, Supplementary Table S2).

In bacteria from biofilms formed following exposure of MAH to DTT, 417 genes (9.7%) were expressed differentially, with 258 genes (6.0%) exhibiting increased expression and 159 genes (3.7%) exhibiting decreased expression (Figure 3C; Supplementary Table S3). Analysis of GO terms referring to biological processes showed significantly enriched expression of genes involved in lipid catabolism, lipid biosynthesis, fatty acid metabolism, DNA recombination, and dicarboxylic acid biosynthesis. Analysis of GO terms referring to molecular functions of gene products indicated significant enrichment of peroxidase activity, inorganic anion transmembrane transport, dioxygenase activity, carbon–oxygen-lyase activity, and 3′-5′ exonuclease activity. GO analysis of cellular components indicated the enrichment of genes involved in encoding ATP-binding cassette (ABC) transporter complexes (Figure 3D, Supplementary Table S4).

**Figure 4:**
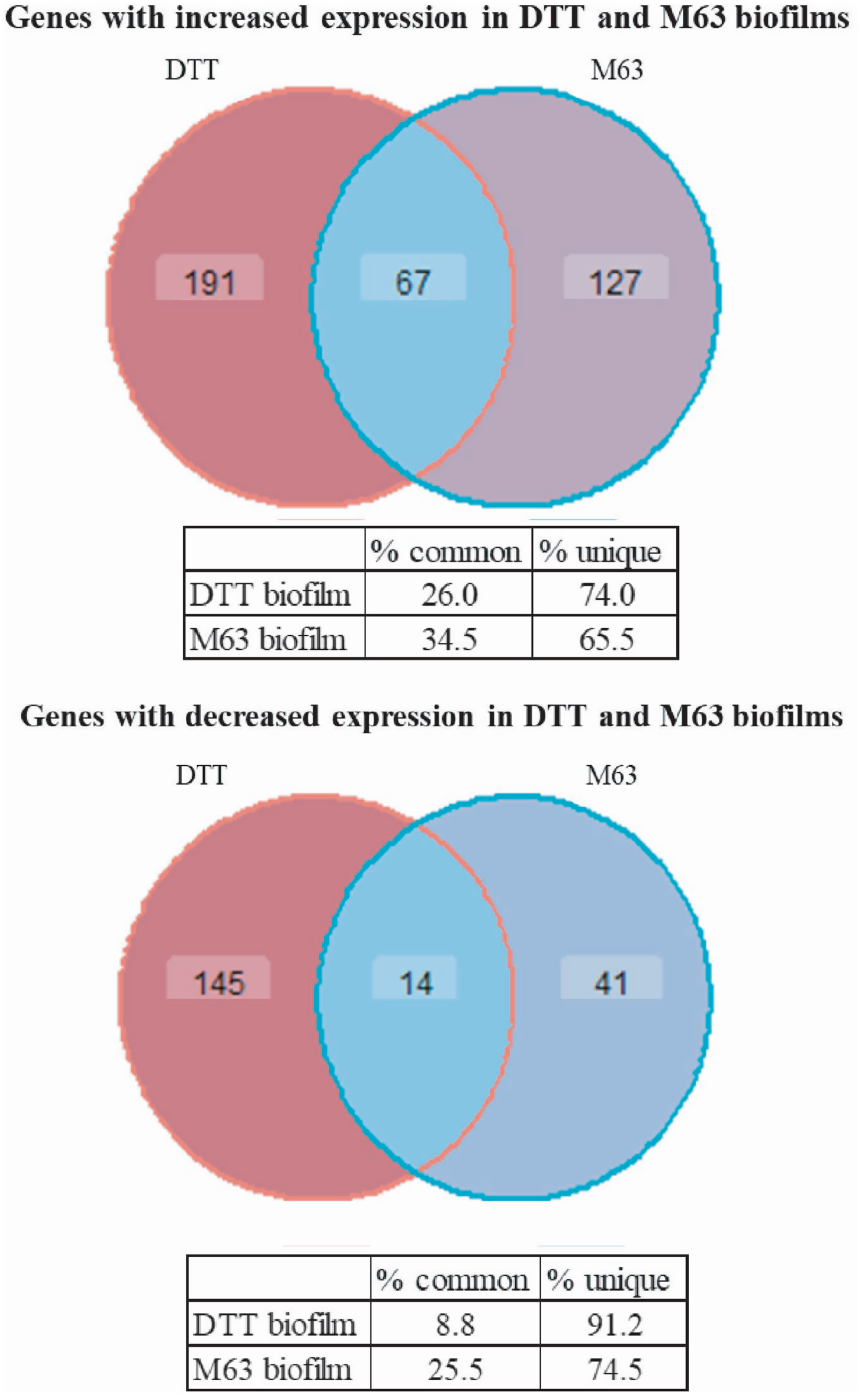
Venn diagram comparison of differentially expressed genes in *M. avium* biofilm models. Significant changes in gene expression relative to planktonic MAH were defined as having a fold-change (log_2_) > 2 and FDR < 0.01 using edgeR. The lists of genes with increased or decreased expression in both models are given in Supplementary Tables S5 and S6, respectively. Venn diagrams are created using ggVennDiagram in R.

Comparing the patterns of DGE between the DTT-induced and M63-incubated biofilm models could reveal potential mechanisms of biofilm formation that are common to both models. In this comparison, the majority of genes with increased or decreased expression relative to planktonic bacteria were differentially expressed in only one biofilm model. However, there were 14 genes with decreased expression and 67 genes with increased expression in both DTT and M63 biofilms (Figure 4, Supplementary Tables S5, S6). MAH in both biofilm models had significant enrichment of genes involved in peroxidase activity based on GO annotations (Supplementary Tables S2, S4). While there were no other commonly enriched GO terms between the biofilm conditions, which is in agreement with the different expression responses seen when analyzing single genes, both conditions clearly stimulated the enrichment of genes involved in multiple metabolic processes, and there may be some overlap in the enriched processes. For example, MAH in the DTT biofilm model had enrichment of genes involved in the metabolism of organic substances (GO:0071704), whereas bacteria in the M63 biofilm model had enrichment of genes involved in the metabolism of organic acids (GO:0006082), which is a direct daughter term of the previous category (Supplementary Tables S2, S4).

### Differential gene expression in MAH during macrophage infection

**Figure 5:**
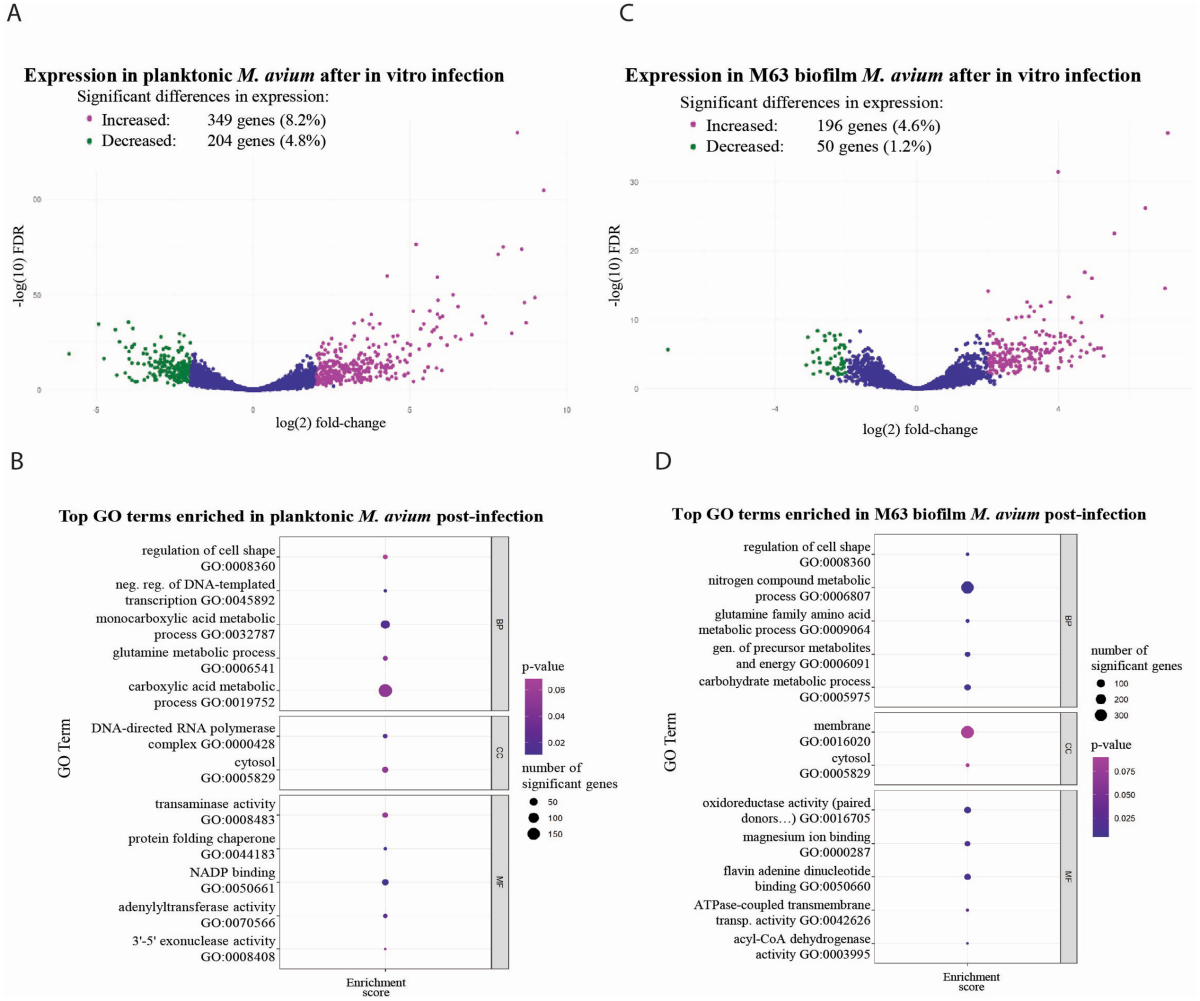
Differential gene expression and GO term enrichment in planktonic and M63 biofilm *M. avium* after 24 h of *in vitro* macrophage infection. Volcano plots showing log_2_ fold expression changes of individual genes in MAH planktonic culture-derived bacteria **(A)** or M63 biofilm culture-derived bacteria **(C)** after a 24-h infection of RAW246.7 macrophages. Significant changes in gene expression were defined as having a fold-change (log_2_) > 2 and FDR < 0.01 using edgeR. Volcano plots were created using edgeR. Dot plots illustrating the top enriched GO terms in MAH post-infection relative to MAH in planktonic culture **(B)** or in M63 biofilm **(D)** prior to infection. The threshold for inclusion was set at weighted Fisher-exact *p* < 0.1. Up to five terms are displayed per category. Dot plots were created using topGO in R. FDR, false discovery rate; GO, gene ontology.

In studies of MAH macrophage infections *in vitro*, it is common to use bacteria derived from a planktonic culture or from a frozen stock that was grown previously in planktonic culture. However, the bacteria that cause infections in susceptible humans originate from environmental sources, where they often persist in biofilms. Since bacteria in liquid culture differ significantly from those in a biofilm, as demonstrated in many previous studies (Rose and Bermudez, 2016; Rose et al., 2015; Trivedi et al., 2016; Chakraborty et al., 2021), and in the RNA-seq comparison of planktonic and biofilm cells described above, we analyzed changes in gene expression that occur when MAH transitions either from planktonic culture or from a biofilm into a macrophage. For this comparison, we used the M63 media, a 4-week incubation biofilm model, which more closely simulates the long-term process of biofilm formation and persistence that occurs in the environment. After a 24-h infection of RAW246.7 macrophages, MAH grown previously in planktonic culture displayed differential expression of 553 genes (12.9%), including 204 genes (4.8%) with decreased expression and 349 genes (8.2%) with increased expression (Figure 5A; Supplementary Table S7). Analysis of GO terms annotating the involvement of genes in biological processes indicated significant enrichment of genes involved in the regulation of cell shape, negative regulation of DNA-templated transcription, and multiple metabolic processes, including carboxylic acid metabolism and glutamine metabolism (Figure 5B, Supplementary Table S8). Analysis of GO terms referring to gene involvement in cellular components showed enrichment of genes involved in the DNA-directed RNA polymerase complex and in the maintenance of the cell cytosol (Figure 5B, Supplementary Table S8). Genes with GO annotations denoting molecular function that were most highly enriched had transaminase activity, NADP binding activity, adenylyltransferase activity, 3′-5′ exonuclease activity, or were protein folding chaperones (Figure 5B).

For MAH isolated from an M63 biofilm, 24-h infection of RAW264.7 macrophages caused differential expression of 246 genes (5.7%), which included 50 genes (1.2%) with decreased expression and 196 genes (4.6%) with increased expression (Figure 5C; Supplementary Table S9). Interestingly, this was approximately half the number of genes that showed DGE between planktonic and macrophage-adapted MAH, suggesting that growth in biofilms may provide some transcriptional benefit for MAH’s adjustment to the macrophage environment. Analysis of GO terms indicating the involvement of genes in specific biological processes showed significant enrichment of genes involved in the regulation of cell shape and multiple metabolic processes, including the generation of precursor metabolites, metabolism of nitrogen compounds, metabolism of carbohydrates, and metabolism of glutamine family amino acids (Figure 5D, Supplementary Table S10). Analysis of GO terms indicating the molecular function of gene products revealed enrichment of genes encoding proteins with acyl-CoA dehydrogenase activity, magnesium ion binding, flavin adenine dinucleotide binding, ATPase-coupled transmembrane transporter activity, and oxidoreductase activity (Figure 5D, Supplementary Table S10).

**Figure 6:**
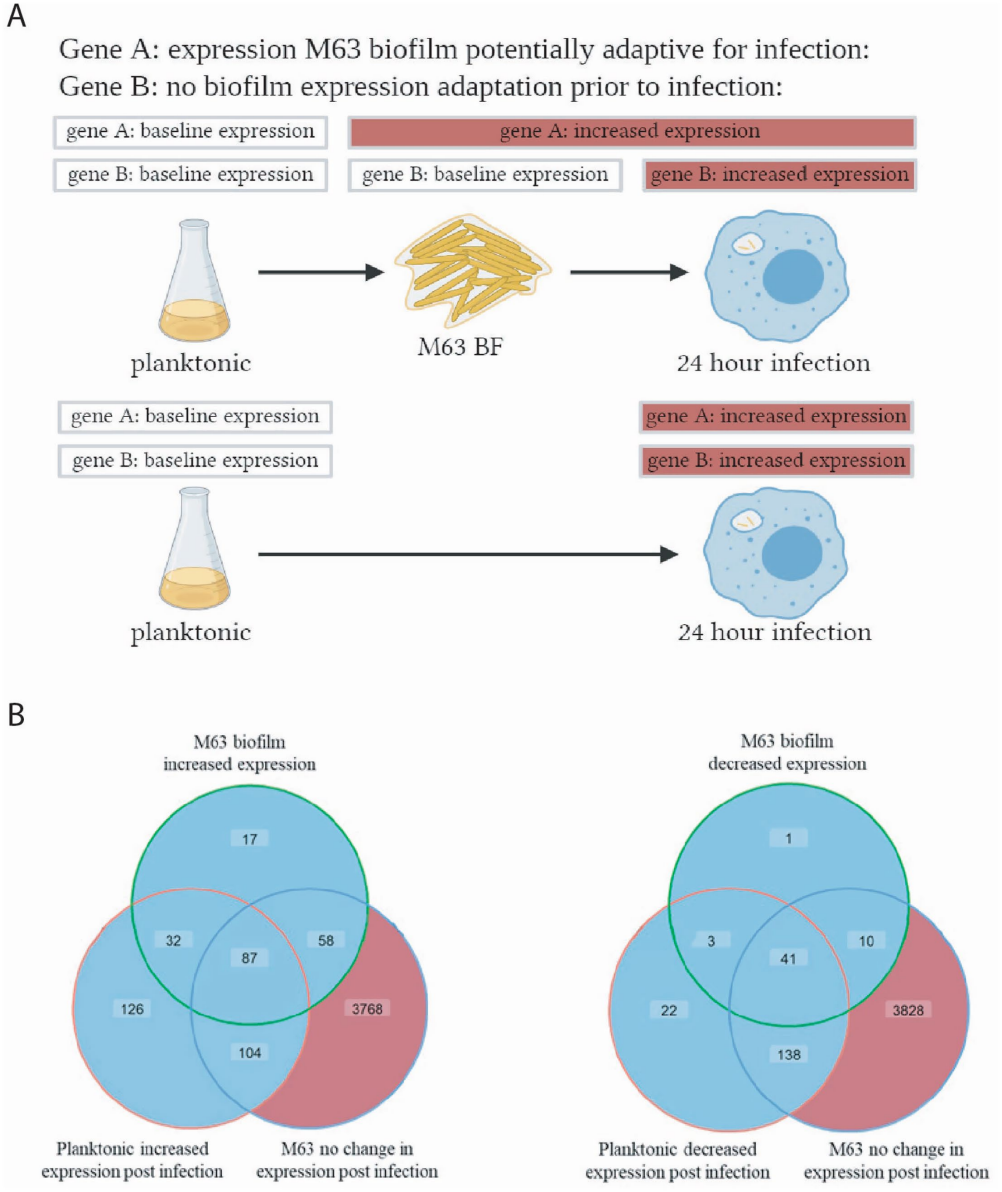
Identification of M63 biofilm gene expression that is potentially adaptive for infection. **(A)** Schematic illustration showing the pattern of increased expression (relative to planktonic MAH) hypothesized to show genes with potentially adaptive patterns of differential expression in M63 biofilms before macrophage infection. Genes following the expression pattern of gene A are captured in the center overlap of the Venn diagram **(B)**. Genes following a similar pattern, but with significantly decreased expression, are captured in the center overlap of the second Venn diagram. Venn diagrams created using ggVennDiagram in R. (**B**) Significant changes in gene expression relative to planktonic MAH were defined as having a fold-change (log_2_) > 2 and FDR < 0.01, and no change in expression was defined as having a fold-change (log_2_) < 2, using edgeR. The lists of genes corresponding to the central area of each Venn diagram are given in Supplementary Tables S11, S12. FDR, false discovery rate.

Because the DGE profiles of the planktonic and M63 biofilm MAH samples isolated post-infection were generated by comparison with different baseline controls (planktonic and M63 biofilm samples, respectively), a direct comparison of the genes differentially expressed in each scenario provides limited information. However, based on our gene expression studies, we hypothesize that the biofilm environment may prepare MAH for survival during infection and that this would be reflected in transcriptional adaptations. As a way to assess this possibility, we identified the sets of genes, when compared to planktonic MAH, that showed significantly altered expression in the MAH isolated from M63 biofilm and planktonic bacteria post-infection but were not differentially expressed when comparing biofilm MAH pre- and post-infection. This suggests that the genes were sufficiently differentially expressed in the nutrient-poor biofilm prior to infection, such that the bacteria no longer required an additional change in gene expression in response to engulfment by macrophages (see schematic illustration Figure 6A). A total of 87 genes showed increased expression and 41 genes showed decreased expression in both M63 biofilm-grown and planktonic-grown MAH post-infection compared to pre-infection planktonic MAH, while also showing no significant change in expression for M63 biofilm-grown MAH pre- and post-macrophage infection (Figure 6B; Supplementary Tables S11, S12). Several GO terms were enriched in these 87 genes, indicating multigene processes and functions that could prepare bacteria in the M63 biofilm to survive during infection. These included significant enrichment for genes involved in cobalamin (vitamin B12) biosynthesis, genes possessing peroxidase activity, and genes with transaminase activity (Supplementary Tables S8, S10).

A comparison of GO terms enriched between the planktonic-derived and M63 biofilm-derived MAH infection trials also revealed commonly enriched biological processes and molecular functions that may indicate responses of the bacteria to infection that occur regardless of the bacteria’s previous growth condition. Biological process GO terms with enrichment in both infection trials included genes involved in the regulation of cell shape, generation of precursor metabolites and energy, and the metabolism of monocarboxylic acid. In addition, genes involved in the metabolism of glutamine family amino acids (GO:0009064) were enriched in M63-derived MAH post-infection, whereas genes annotated with the closely related metabolism of glutamine (GO:0006541) were enriched in planktonic-derived MAH post-infection (Supplementary Tables S8, S10). GO terms associated with the molecular function annotated to gene products that were enriched in both infection trials included genes with oxidoreductase activity (acting on paired donors, with incorporation or reduction of molecular oxygen) and phosphotransferase activity (alcohol group as an acceptor) (Supplementary Tables S8, S10).

### Validation by qRT-PCR

To validate the changes observed in the RNA-seq data, we performed qRT-PCR on several genes, which showed increased expression in planktonic MAH after macrophage infection and M63 biofilm-grown MAH compared to planktonic MAH, but which were not further differentially expressed when M63 biofilm MAH underwent macrophage infection. We validated three genes: *tetR*, an important transcriptional regulator in mycobacteria (Abukhalid et al., 2023; Liu et al., 2016; Liu et al., 2016), and two PPE genes, a class of genes enriched in our pool of upregulated genes in biofilms and macrophage infections. We observed similar patterns of expression changes using qRT-PCR and RNA-seq for all three genes, although the magnitude of changes differed between methods in some cases. All three genes exhibited upregulation in M63 biofilms relative to planktonic MAH and increased expression in planktonic-grown MAH from infected macrophages relative to planktonic MAH, but not relative to M63 biofilm MAH (Table 1). Although not tested in the RNA-seq analysis, MAH from DTT-based biofilms showed the same trends as observed in the M63 biofilm MAH: increased gene expression in biofilm relative to planktonic, but no change in gene expression pre- and post-infection when using the DTT biofilm MAH. This suggests that MAH, grown even under different initial biofilm conditions, undergoes similar transcriptional changes that may promote the mycobacteria’s adaptation to the macrophage environment.

**Table 1:** Differential gene expression by qRT-PCR or RNAseq for *M. avium* cultured under different growth conditions or pre- or post-infection.

Fold change	TetR		RS22425		RS22460	
	qPCR	RNAseq	qPCR	RNAseq	qPCR	RNAseq
M63_Biofilm rel. Plank	276.32	4.99	89.27	41.07	35.23	5.80
DTT Biofilm rel. Plank	68.48	4.66	51.75	47.34	26.69	32.45
Inf. A5 rel. Plank	14.71	2.69	8.42	22.94	9.64	9.08
Inf. M63 rel. M63 Biofilm	0.67	1.44	0.94	0.74	NA	2.83
Inf. DTT rel. DTT Biofilm	0.31	NA	0.06	NA	0.09	NA

## Discussion

The remarkable ability of MAH to persist through environmental stress and exposure to bactericidal agents has led to the hypothesis that life in a biofilm causes adaptations that enable the bacterium to survive and establish an infection when inhaled (Chakraborty and Kumar, 2019). Studies modeling infection and colonization of the bronchiolar and bronchial epithelium by *M. avium* have shown that the ability of *M. avium* to form biofilms is correlated with its ability to invade respiratory epithelial cells and establish pulmonary infections in mice (Yamazaki et al., 2006a; Yamazaki et al., 2006b). In these studies, disruption of several genes, including those encoding enzymes involved in GPL biosynthesis and the TCA cycle, attenuated the ability of *M. avium* to form biofilms and to establish pulmonary infection in mice (Yamazaki et al., 2006a; Yamazaki et al., 2006b). Although these studies suggest common genetic determinants of biofilm formation and virulence, there has been no comparison of gene expression between these features.

In our RNA-seq study, we found that, relative to bacteria in planktonic culture, multiple genes in MAH were expressed at higher levels in both biofilm models and during macrophage infection, relative to planktonic MAH, suggesting that these genes may be important for the bacterium’s response to a variety of stress conditions. Of the five universal stress proteins (USPs) identified in the MAH genome, four (BJP76_RS10615, BJP76_RS17850, BJP76_RS10620, and BJP76_RS10555) had significantly increased expression in both biofilm models and during infection. This class of proteins is widespread across bacteria, archaea, fungi, and plants. Although characterization of USPs in mycobacteria has been limited, most of the 10 USPs in *M. tuberculosis* are induced by multiple stresses, including hypoxia, low pH, and nitric oxide, leading to the hypothesis that USP expression may be important for persistence in vivo (Rimal et al., 2023). In *M. tuberculosis*, mutation of single USP genes appears to have no effect on the persistence of the bacterium under various stress conditions, suggesting that there may be functional redundancies in the proteins encoded by this group of genes (Hingley-Wilson et al., 2010). We also observed differential expression of numerous PE/PPE family proteins, including eight PPE proteins (BJP76_RS06060, BJP76_RS05340, BJP76_RS10270, BJP76_RS22460, BJP76_RS11805, BJP76_RS11800, BJP76_RS22425, and BJP76_RS08365) and one PE protein (BJP76_RS05345), with increased expression in both biofilm conditions and during macrophage infection. These proteins, named for their highly conserved N-terminal proline-glutamic acid (PE) or proline-proline-glutamic acid (PPE) motifs, are localized to the mycobacterial membrane or secreted and are implicated in a variety of virulence-related activities in *M. tuberculosis*, including stress resistance, modulation of immune cells and inflammatory responses, and resistance to drugs (D'Souza et al., 2023). Little is known about PE/PPE protein functions in *M. avium* (Mackenzie et al., 2009). Within the PE/PPE family, there is a high level of variability in the C-termini of the proteins and low conservation of sequences between species (Marri et al., 2006), making it difficult to identify possible orthologs with confidence. One PPE protein gene that had increased expression under biofilm and infection conditions (BJP76_RS22425, previously annotated as MAV_0118) has been observed to have increased expression in *M. avium* during infection in mice (Ignatov et al., 2010) and human macrophages (Hou et al., 2002), supporting the hypothesis that this protein plays a role in persistence during infection. Further functional studies of both USPs and PE/PPE proteins in MAH are needed to better understand the importance of these proteins in facilitating the bacterial response to a variety of stressors.Other genes with increased expression under biofilm conditions and during infection had more clearly identified functions. While there was limited overlap in enriched GO terms between the conditions studied, all conditions had enriched GO terms related to redox homeostasis, and some of the genes that had increased expression in biofilms and during infection appeared to be involved in redox homeostasis. Expression of the gene encoding the hydrogen peroxide-inducible gene activator OxyR (BJP76_RS11430) increased significantly under both biofilm conditions and during infection of macrophages. OxyR is the central regulator of the bacterial oxidative stress response and is conserved across many species. In *M. avium*, increased expression of *oxyR* has been observed in response to oxidative stress caused by autoinducer 2 (Geier et al., 2008). Interestingly, *oxyR* is not functional in *M. tuberculosis*; therefore, the regulation of genes by OxyR in response to stress may be a major distinction between the behavior of MAH and *M. tuberculosis* during infection (Pagan-Ramos et al., 2006).

Other changes in gene expression indicate changes in metabolic processes, which were prominently featured in the GO enrichment analyses in both biofilm and infection conditions, although each condition seemed to induce responses in different metabolic pathways, with only some observed overlap. However, the increased expression of certain genes involved in the biosynthesis or catabolism of various molecules was observed in MAH in both biofilm models and during macrophage infection. BJP76_RS18340 encodes a pyridoxamine 5′-phosphate oxidase family protein involved in the synthesis of pyridoxal 5-phosphate, which has previously been observed to be essential for survival and virulence of *M. tuberculosis* (Dick et al., 2010). Another gene, *metH* (BJP76_RS09995), encoding methionine synthase, showed significantly increased expression in DTT biofilms and during infection of macrophages and mildly increased expression in M63 biofilms. *MetE* (BJP76_RS05740), which catalyzes the transfer of a methyl group from 5-methyltetrahydrofolate to homocysteine, resulting in methionine formation, was also increased under all conditions, although slightly below the significance threshold in *M. avium* during infection. In *M. tuberculosis*, biosynthesis of methionine and the closely related S-adenosyl methionine (SAM) are essential for the survival of the pathogen *in vivo* (Berney et al., 2015), and increased *metH* expression has been observed previously in *M. avium* in the context of infection in mice (Ignatov et al., 2010). Two genes involved in the metabolism of carbohydrates were also upregulated significantly, or nearly significantly, in all conditions. BJP76_RS04430 encodes a glycogen debranching N-terminal domain-containing protein, and BJP76_RS04425 encodes a glycosyltransferase family 4 protein. While there are many possible specific activities that the products of these genes could be involved in, such as liberation of glucose for use as an energy source and glycosylation of a general category of proteins, respectively, it is intriguing to consider the possibility that these enzymes could play a role in cellulose synthesis, which occurs in biofilms (Trivedi et al., 2016; Chakraborty et al., 2021) and during *in vivo* infection (Chakraborty et al., 2021), but which is accomplished by a presently unknown mechanism.

Many genes that had increased expression in both biofilm models also had increased expression during infection, as discussed above. However, there were also several genes that had increased expression in both biofilm models but were not differentially expressed during infection, indicating that these genes may have roles that are of specific importance for biofilm formation. These included additional PE/PPE family proteins (BJP76_RS06030 and BJP76_RS22405). Another gene with expression increased exclusively in biofilms was an acyl-ACP desaturase (BJP76_RS08205), which is an ortholog of the *M. tuberculosis* gene *desA1* (75% amino acid sequence identity, 86% amino acid sequence similarity). In *M. smegmatis*, DesA1 has been shown to play an essential role in the biosynthesis of mycolic acids, the primary component of the mycobacterial cell wall, and is hypothesized to have a similar role in *M. tuberculosis* (Singh et al., 2016; Yeruva et al., 2016). Furthermore, *desA1* is necessary for the viability of *M. tuberculosis in vitro* (Pagan-Ramos et al., 2006), and its Ca^2+^-binding structural feature has made it a target of interest for drug development (Yeruva et al., 2016).

In addition, a TetR/AcrR family transcriptional regulator (BJP76_RS10680) showed increased expression in both biofilm models. This specific protein does not have a close ortholog in *M. tuberculosis*, but the TetR/AcrR family transcription regulators have been implicated in drug resistance, pathogenicity, and response to stress in multiple species of bacteria (Ramos et al., 2005). *M. avium* also had significantly increased expression of the thiol reductant ATP-Binding Cassette (ABC) exporter subunit *cydD* gene (BJP76_RS13220) in both biofilm conditions tested. The *E. coli* ortholog of the protein encoded by this gene has been shown to facilitate the export of cysteine and glutathione from bacterial cells (Pittman et al., 2002; Pittman et al., 2005), indicating a potential role in bacterial response to reducing stressors like glutathione and DTT, and the *M. tuberculosis* ortholog participates in the electron transport chain (Barreto et al., 2020). Together, it appears that *cydD* and its associated genes may have multiple functions, and further studies characterizing their role in response to biofilm conditions in *M. avium* would be informative.

The application of RNA-seq in this study is the first use of this powerful NGS tool for comparing gene expression in MAH under different conditions, and it has provided many possibilities for further study. In our approach, we pooled all RNA from all bacteria within a given condition at a set time point in order to identify changes in gene expression that are broadly characteristic of MAH during growth or survival in a biofilm or in a macrophage. One limitation of this approach is that gene expression in each condition was characterized at a single time point, making it impossible to observe the dynamics and timing of the expression changes detected and causing us to miss changes in expression that undoubtedly occur earlier or later than each of our chosen time points. However, the single time points characterized in each condition in our study yielded novel information about MAH and its transcriptional responses, and the dynamics of gene expression changes detected in our study can be characterized in the future by further experiments using qRT-PCR. Another limitation of our RNA-seq approach is that by pooling all bacteria within a given condition, we were unable to assess variation in gene expression among bacteria within a given condition. In planktonic cultures, bacteria experience similar conditions, leading to relatively homogenous patterns of gene expression. However, biofilm communities contain bacteria with various phenotypes due, at least in part, to differences in gene expression, coordinating to enhance survival (Flemming et al., 2016; Richards and Ojha, 2014). The range of phenotypes present in biofilms of MAH and other mycobacteria has not yet been assessed. Future studies on MAH biofilms could characterize the heterogeneous gene expression patterns present within a biofilm using single-cell RNA-seq technology. Although single-cell RNA-seq has not been used to characterize mycobacteria previously, recent improvements in optimizing the technology for studying bacteria would facilitate this type of study (Ma et al., 2023). This could even be used to study gene expression in environmental isolates from different environments, enabling the direct characterization of gene expression patterns that allow MAH to persist in a wide variety of environmental niches.

## Data Availability

The original contributions presented in the study are publicly available. This data can be found here: https://www.ncbi.nlm.nih.gov/, accession number PRJNA1365422.
