# Supplementary material for: Growth in biofilms prepares Mycobacterium avium subsp. hominissuis for the macrophage microenvironment
Source: Front Microbiol. 2025 Dec 4;16:1709239. doi: 10.3389/fmicb.2025.1709239 (PMC12711761; doi:10.3389/fmicb.2025.1709239)
Supplement: Supplementary file 1 [file Table_1.DOCX]

Supplementary Tables

TABLE S1: Top 30 genes with greatest expression increase and bottom 30 genes with greatest expression decrease in M63 biofilm *M. avium*, relative to planktonic

| **Symbol** | **Description** | **log2FC** | **logCPM** | **PValue** | **FDR** |
| --- | --- | --- | --- | --- | --- |
| BJP76_RS11805 | PPE family protein | 8.940 | 8.750 | 2.24E-103 | 9.61E-100 |
| BJP76_RS11800 | PPE family protein | 7.828 | 8.411 | 9.01E-68 | 1.29E-64 |
| BJP76_RS13530 | group 1 truncated hemoglobin | 7.426 | 6.955 | 6.49E-28 | 1.46E-25 |
| BJP76_RS14240 | methyltransferase domain-containing protein | 6.960 | 8.826 | 9.59E-18 | 8.92E-16 |
| BJP76_RS08730 | bifunctional nitrate reductase/sulfite reductase flavoprotein subunit alpha | 6.823 | 9.760 | 2.70E-101 | 5.79E-98 |
| BJP76_RS11795 | DUF732 domain-containing protein | 6.779 | 7.052 | 1.68E-36 | 8.98E-34 |
| BJP76_RS05345 | PE domain-containing protein | 6.455 | 9.692 | 2.79E-32 | 7.45E-30 |
| BJP76_RS22550 | nitrate/nitrite transporter | 6.130 | 7.795 | 8.38E-41 | 5.12E-38 |
| BJP76_RS18340 | pyridoxamine 5'-phosphate oxidase family protein | 6.019 | 8.961 | 2.28E-10 | 7.73E-09 |
| BJP76_RS11790 | DUF732 domain-containing protein | 5.943 | 5.572 | 2.80E-64 | 3.00E-61 |
| BJP76_RS22555 | uroporphyrinogen-III synthase | 5.607 | 7.243 | 1.22E-42 | 8.68E-40 |
| BJP76_RS10615 | universal stress protein | 5.602 | 11.243 | 5.60E-12 | 2.37E-10 |
| BJP76_RS05340 | PPE family protein | 5.597 | 9.977 | 4.07E-26 | 8.30E-24 |
| BJP76_RS00835 | NAD(P)/FAD-dependent oxidoreductase | 5.381 | 9.977 | 2.72E-11 | 1.06E-09 |
| BJP76_RS22425 | PPE family protein | 5.360 | 7.393 | 3.41E-34 | 1.46E-31 |
| BJP76_RS22560 | sirohydrochlorin chelatase | 5.126 | 7.028 | 4.01E-28 | 9.54E-26 |
| BJP76_RS15910 | PPE family protein | 4.995 | 7.014 | 2.89E-31 | 7.28E-29 |
| BJP76_RS10270 | PPE family protein | 4.967 | 8.672 | 1.72E-32 | 5.65E-30 |
| BJP76_RS22575 | nitrite reductase large subunit NirB | 4.875 | 10.532 | 1.02E-33 | 3.96E-31 |
| BJP76_RS13570 | Rv1535 family protein | 4.837 | 7.244 | 4.09E-21 | 5.31E-19 |
| BJP76_RS06060 | PPE domain-containing protein | 4.790 | 5.193 | 2.28E-25 | 4.25E-23 |
| BJP76_RS14890 | hemerythrin domain-containing protein | 4.539 | 6.368 | 4.46E-15 | 2.98E-13 |
| BJP76_RS10620 | universal stress protein | 4.389 | 11.885 | 4.94E-07 | 7.48E-06 |
| BJP76_RS05125 | PPE family protein | 4.313 | 8.231 | 5.63E-15 | 3.71E-13 |
| BJP76_RS11825 | PE family protein | 4.253 | 5.301 | 2.30E-06 | 2.96E-05 |
| BJP76_RS22570 | nitrite reductase small subunit NirD | 4.183 | 8.155 | 2.76E-18 | 2.88E-16 |
| BJP76_RS15905 | hypothetical protein | 4.152 | 4.286 | 3.25E-16 | 2.49E-14 |
| BJP76_RS22580 | Hsp20/alpha crystallin family protein | 4.093 | 8.911 | 5.97E-14 | 3.41E-12 |
| BJP76_RS00830 | TetR/AcrR family transcriptional regulator | 4.078 | 6.332 | 3.61E-12 | 1.56E-10 |
| BJP76_RS21790 | DUF1800 domain-containing protein | 4.040 | 5.296 | 7.39E-23 | 1.13E-20 |
| BJP76_RS21715 | hypothetical protein | -2.515 | 4.034 | 4.27E-14 | 2.54E-12 |
| BJP76_RS11780 | PPE family protein | -2.523 | 6.091 | 2.24E-07 | 3.71E-06 |
| BJP76_RS11970 | enoyl-CoA hydratase-related protein | -2.523 | 5.142 | 8.96E-16 | 6.61E-14 |
| BJP76_RS03890 | ferredoxin | -2.547 | 3.307 | 2.41E-06 | 3.07E-05 |
| BJP76_RS05805 | aldehyde dehydrogenase family protein | -2.551 | 6.187 | 3.06E-13 | 1.54E-11 |
| BJP76_RS18105 | alpha/beta hydrolase | -2.552 | 6.732 | 2.77E-06 | 3.49E-05 |
| BJP76_RS00670 | hypothetical protein | -2.558 | 6.365 | 2.84E-09 | 8.06E-08 |
| BJP76_RS00740 | WXG100 family type VII secretion target | -2.574 | 9.865 | 6.40E-06 | 7.09E-05 |
| BJP76_RS10845 | MPT63 family protein | -2.599 | 4.958 | 1.35E-07 | 2.46E-06 |
| BJP76_RS16560 | HU family DNA-binding protein | -2.694 | 12.015 | 3.22E-09 | 8.94E-08 |
| BJP76_RS12865 | hypothetical protein | -2.768 | 6.455 | 8.24E-08 | 1.58E-06 |
| BJP76_RS17685 | fluoride efflux transporter CrcB | -2.791 | 4.226 | 4.47E-07 | 6.79E-06 |
| BJP76_RS11775 | PPE family protein | -2.806 | 5.383 | 3.68E-08 | 7.72E-07 |
| BJP76_RS00190 | acyl-CoA dehydrogenase family protein | -2.816 | 6.182 | 1.61E-05 | 1.54E-04 |
| BJP76_RS14140 | DUF732 domain-containing protein | -2.856 | 2.759 | 6.18E-04 | 3.30E-03 |
| BJP76_RS23665 | aldehyde dehydrogenase family protein | -2.883 | 6.468 | 4.82E-08 | 9.73E-07 |
| BJP76_RS06645 | NUDIX hydrolase | -2.889 | 5.709 | 2.03E-22 | 2.90E-20 |
| BJP76_RS18905 | NnrU family protein | -2.903 | 6.712 | 4.38E-10 | 1.42E-08 |
| BJP76_RS16030 | MPT63 family protein | -2.972 | 6.209 | 6.37E-09 | 1.65E-07 |
| BJP76_RS18145 | Rho termination factor N-terminal domain-containing protein | -3.137 | 4.733 | 2.15E-04 | 1.39E-03 |
| BJP76_RS06630 | tetratricopeptide repeat protein | -3.159 | 7.480 | 9.13E-08 | 1.72E-06 |
| BJP76_RS18100 | flavin monoamine oxidase family protein | -3.279 | 6.408 | 1.04E-06 | 1.47E-05 |
| BJP76_RS01260 | lipoprotein LpqH | -3.468 | 7.665 | 1.90E-13 | 9.66E-12 |
| BJP76_RS20780 | LpqN/LpqT family lipoprotein | -3.552 | 6.148 | 6.37E-14 | 3.52E-12 |
| BJP76_RS13195 | hypothetical protein | -3.558 | 7.134 | 2.05E-07 | 3.47E-06 |
| BJP76_RS11770 | DUF732 domain-containing protein | -3.672 | 4.464 | 2.97E-08 | 6.42E-07 |
| BJP76_RS00195 | WhiB family transcriptional regulator | -3.864 | 6.210 | 1.89E-05 | 1.79E-04 |
| BJP76_RS21720 | hypothetical protein | -3.896 | 5.725 | 3.10E-20 | 3.68E-18 |
| BJP76_RS20900 | SulP family inorganic anion transporter | -4.262 | 9.748 | 4.17E-18 | 4.06E-16 |
| BJP76_RS06650 | DUF732 domain-containing protein | -4.430 | 7.061 | 9.35E-12 | 3.85E-10 |

TABLE S2: top go terms enriched in *M. avium* in M63 biofilms

|  | **GO.ID** | **Term** | **Annotated genes** | **Significant DE genes (FDR <0.05)** | **Expected DE genes** | **Weighted Fisher-exact p-value** |
| --- | --- | --- | --- | --- | --- | --- |
| Biological process (BP) | GO:1901615 | organic hydroxy compound metabolic process | 52 | 25 | 22.7 | 0.036 |
|  | GO:0009236 | cobalamin biosynthetic process | 13 | 9 | 5.7 | 0.057 |
|  | GO:0006541 | glutamine metabolic process | 21 | 13 | 9.2 | 0.070 |
|  | GO:0006082 | organic acid metabolic process | 298 | 138 | 130.0 | 0.081 |
|  | GO:0019725 | cellular homeostasis | 13 | 7 | 5.7 | 0.083 |
|  | GO:0006313 | DNA transposition | 54 | 29 | 23.6 | 0.085 |
|  | GO:0009201 | ribonucleoside triphosphate biosynthetic process | 10 | 7 | 4.4 | 0.086 |
| Molecular function (MF) | GO:0016884 | carbon-nitrogen ligase activity, with glutamine as amido-N-donor | 14 | 12 | 6.1 | 0.002 |
|  | GO:1901681 | sulfur compound binding | 25 | 16 | 11.0 | 0.004 |
|  | GO:0016661 | oxidoreductase activity, acting on other nitrogenous compounds as donors | 10 | 8 | 4.4 | 0.023 |
|  | GO:0004601 | peroxidase activity | 35 | 20 | 15.3 | 0.070 |
|  | GO:0004803 | transposase activity | 53 | 29 | 23.2 | 0.070 |
|  | GO:0016866 | intramolecular transferase activity | 21 | 13 | 9.2 | 0.073 |
|  | GO:0008483 | transaminase activity | 27 | 16 | 11.8 | 0.077 |
|  | GO:0016705 | oxidoreductase activity, acting on paired donors, with incorporation or reduction of molecular oxygen | 146 | 72 | 63.9 | 0.098 |

TABLE S3: Top 30 genes with greatest expression increase and bottom 30 genes with greatest expression decrease in DTT biofilm *M. avium*, relative to planktonic

| **Symbol** | **Description** | **log2FC** | **logCPM** | **PValue** | **FDR** |
| --- | --- | --- | --- | --- | --- |
| BJP76_RS10615 | universal stress protein | 9.788 | 11.243 | 2.56E-25 | 2.39E-23 |
| BJP76_RS18340 | pyridoxamine 5'-phosphate oxidase family protein | 9.116 | 8.961 | 4.50E-18 | 1.48E-16 |
| BJP76_RS17845 | group III truncated hemoglobin | 9.026 | 10.539 | 1.41E-22 | 8.75E-21 |
| BJP76_RS17850 | universal stress protein | 8.997 | 12.084 | 1.77E-13 | 2.71E-12 |
| BJP76_RS12375 | DUF4383 domain-containing protein | 8.819 | 11.406 | 1.22E-49 | 7.48E-47 |
| BJP76_RS10620 | universal stress protein | 8.678 | 11.885 | 1.19E-17 | 3.57E-16 |
| BJP76_RS10605 | hypothetical protein | 8.521 | 10.323 | 5.82E-28 | 8.03E-26 |
| BJP76_RS11430 | hydrogen peroxide-inducible genes activator | 8.148 | 6.810 | 2.16E-19 | 8.40E-18 |
| BJP76_RS10595 | nitroreductase family protein | 7.530 | 10.438 | 6.39E-16 | 1.44E-14 |
| BJP76_RS08335 | HSP20 family small heat-shock protein | 7.301 | 8.501 | 1.58E-14 | 2.86E-13 |
| BJP76_RS10555 | universal stress protein | 7.241 | 11.760 | 4.30E-16 | 1.01E-14 |
| BJP76_RS10915 | helix-turn-helix transcriptional regulator | 7.024 | 7.120 | 9.36E-15 | 1.75E-13 |
| BJP76_RS04430 | glycogen debranching N-terminal domain-containing protein | 7.009 | 8.564 | 6.62E-16 | 1.48E-14 |
| BJP76_RS10625 | PAS domain S-box protein | 6.988 | 10.483 | 2.08E-24 | 1.68E-22 |
| BJP76_RS10610 | nitroreductase family protein | 6.961 | 11.161 | 4.56E-13 | 6.40E-12 |
| BJP76_RS10600 | STAS/SEC14 domain-containing protein | 6.927 | 9.868 | 2.12E-26 | 2.26E-24 |
| BJP76_RS08340 | hypothetical protein | 6.850 | 7.345 | 6.69E-15 | 1.30E-13 |
| BJP76_RS12380 | DUF2510 domain-containing protein | 6.672 | 9.941 | 2.28E-61 | 2.44E-58 |
| BJP76_RS05345 | PE domain-containing protein | 6.324 | 9.692 | 4.46E-33 | 8.68E-31 |
| BJP76_RS04425 | glycosyltransferase family 4 protein | 6.206 | 8.088 | 9.52E-17 | 2.46E-15 |
| BJP76_RS18345 | nitroreductase family protein | 6.046 | 8.316 | 1.43E-08 | 9.72E-08 |
| BJP76_RS05340 | PPE family protein | 5.903 | 9.977 | 6.40E-30 | 9.44E-28 |
| BJP76_RS23750 | sodium:proton exchanger | 5.734 | 7.893 | 3.04E-27 | 4.07E-25 |
| BJP76_RS10270 | PPE family protein | 5.733 | 8.672 | 5.10E-43 | 2.18E-40 |
| BJP76_RS21595 | hypothetical protein | 5.616 | 6.829 | 1.97E-16 | 4.86E-15 |
| BJP76_RS22425 | PPE family protein | 5.565 | 7.393 | 1.51E-38 | 4.03E-36 |
| BJP76_RS08775 | dimethylarginine dimethylaminohydrolase family protein | 5.483 | 6.590 | 4.68E-31 | 8.02E-29 |
| BJP76_RS08320 | transcription elongation factor GreA | 5.381 | 5.180 | 5.26E-65 | 1.13E-61 |
| BJP76_RS13220 | thiol reductant ABC exporter subunit CydD | 5.352 | 9.116 | 3.61E-34 | 8.13E-32 |
| BJP76_RS10920 | HSP20 family small heat-shock protein | 5.332 | 8.343 | 1.63E-10 | 1.54E-09 |
| BJP76_RS16565 | 3-isopropylmalate dehydratase small subunit LeuD | -3.215 | 7.941 | 1.07E-07 | 6.03E-07 |
| BJP76_RS18235 | PfkB family carbohydrate kinase | -3.222 | 2.238 | 7.73E-09 | 5.54E-08 |
| BJP76_RS10035 | MCE family protein | -3.243 | 3.998 | 2.77E-17 | 7.95E-16 |
| BJP76_RS08465 | hypothetical protein | -3.254 | 6.760 | 4.55E-31 | 8.02E-29 |
| BJP76_RS07070 | ABC transporter ATP-binding protein | -3.292 | 6.148 | 1.48E-10 | 1.42E-09 |
| BJP76_RS23845 | OB-fold domain-containing protein | -3.334 | 1.899 | 6.51E-14 | 1.09E-12 |
| BJP76_RS21890 | histidine phosphatase family protein | -3.340 | 4.678 | 1.57E-24 | 1.32E-22 |
| BJP76_RS22310 | Hsp70 family protein | -3.344 | 7.127 | 3.61E-22 | 2.06E-20 |
| BJP76_RS07575 | DMT family transporter | -3.360 | 6.125 | 2.40E-20 | 1.05E-18 |
| BJP76_RS08425 | alpha/beta fold hydrolase | -3.371 | 4.052 | 7.67E-08 | 4.49E-07 |
| BJP76_RS00430 | SDR family oxidoreductase | -3.403 | 4.867 | 9.88E-27 | 1.14E-24 |
| BJP76_RS10045 | ABC transporter permease | -3.407 | 4.605 | 2.88E-12 | 3.60E-11 |
| BJP76_RS00145 | long-chain-fatty acid--ACP ligase MbtM | -3.456 | 4.661 | 2.42E-10 | 2.23E-09 |
| BJP76_RS08420 | non-ribosomal peptide synthetase | -3.476 | 7.259 | 2.71E-13 | 3.96E-12 |
| BJP76_RS02025 | DUF4193 domain-containing protein | -3.533 | 4.642 | 1.38E-19 | 5.46E-18 |
| BJP76_RS16030 | MPT63 family protein | -3.585 | 6.209 | 3.30E-17 | 9.34E-16 |
| BJP76_RS08200 | salicylate synthase | -3.651 | 6.650 | 4.54E-12 | 5.46E-11 |
| BJP76_RS08430 | polyketide synthase | -3.803 | 5.553 | 7.16E-11 | 7.29E-10 |
| BJP76_RS14140 | DUF732 domain-containing protein | -3.849 | 2.759 | 6.13E-08 | 3.65E-07 |
| BJP76_RS08460 | hypothetical protein | -3.952 | 8.511 | 1.37E-26 | 1.50E-24 |
| BJP76_RS13400 | mechanosensitive ion channel family protein | -4.014 | 5.929 | 8.98E-27 | 1.07E-24 |
| BJP76_RS04610 | Nramp family divalent metal transporter | -4.119 | 6.292 | 3.31E-17 | 9.34E-16 |
| BJP76_RS18205 | glycosyltransferase family 2 protein | -4.228 | 2.030 | 4.87E-10 | 4.23E-09 |
| BJP76_RS08435 | mycobactin polyketide synthase MbtD | -4.238 | 6.437 | 1.80E-14 | 3.22E-13 |
| BJP76_RS08450 | NADPH-dependent L-lysine N(6)-monooxygenase MbtG | -4.385 | 6.006 | 2.49E-20 | 1.08E-18 |
| BJP76_RS08440 | non-ribosomal peptide synthetase | -4.395 | 8.482 | 4.30E-15 | 8.56E-14 |
| BJP76_RS11940 | ANTAR domain-containing protein | -4.439 | 7.046 | 3.77E-37 | 8.98E-35 |
| BJP76_RS08445 | non-ribosomal peptide synthetase | -4.616 | 7.296 | 4.07E-17 | 1.12E-15 |
| BJP76_RS17010 | hypothetical protein | -4.720 | 6.124 | 6.05E-30 | 9.25E-28 |
| BJP76_RS11345 | transglycosylase family protein | -4.828 | 5.854 | 1.13E-39 | 4.03E-37 |

TABLE S4: Top go terms enriched in *M. avium* in DTT biofilms

|  | **GO.ID** | **Term** | **Annotated genes** | **Significant DE genes (FDR <0.05)** | **Expected DE genes** | **Weighted Fisher-exact p-value** |
| --- | --- | --- | --- | --- | --- | --- |
| Biological process (BP) | GO:0006631 | fatty acid metabolic process | 66 | 45 | 40.2 | 0.009 |
|  | GO:0008610 | lipid biosynthetic process | 94 | 61 | 57.2 | 0.012 |
|  | GO:0016042 | lipid catabolic process | 23 | 19 | 14.0 | 0.022 |
|  | GO:0006310 | DNA recombination | 79 | 55 | 48.1 | 0.034 |
|  | GO:0043650 | dicarboxylic acid biosynthetic process | 21 | 17 | 12.8 | 0.043 |
|  | GO:0072330 | monocarboxylic acid biosynthetic process | 38 | 26 | 23.1 | 0.065 |
|  | GO:0044283 | small molecule biosynthetic process | 206 | 130 | 125.4 | 0.078 |
|  | GO:0071704 | organic substance metabolic process | 1276 | 772 | 776.5 | 0.088 |
|  | GO:0006259 | DNA metabolic process | 159 | 105 | 96.8 | 0.088 |
| Cellular component (CC) | GO:0043190 | ATP-binding cassette (ABC) transporter complex | 27 | 20 | 16.2 | 0.090 |
| Molecular function (MF) | GO:0016835 | carbon-oxygen lyase activity | 85 | 53 | 51.9 | 0.007 |
|  | GO:0008408 | 3'-5' exonuclease activity | 10 | 9 | 6.1 | 0.053 |
|  | GO:0004601 | peroxidase activity | 35 | 27 | 21.4 | 0.081 |
|  | GO:0051213 | dioxygenase activity | 71 | 50 | 43.4 | 0.094 |
|  | GO:0015103 | inorganic anion transmembrane transporter activity | 12 | 10 | 7.3 | 0.095 |
|  | GO:0016833 | oxo-acid-lyase activity | 12 | 10 | 7.3 | 0.095 |

TABLE S5: *M. avium* genes with increased expression in both M63 and DTT in vitro biofilm models

| **Symbol** | **Description** | **DTT log2FC** | **M63 log2FC** |
| --- | --- | --- | --- |
| BJP76_RS01245 | two-component system response regulator TcrX | 2.154 | 2.410 |
| BJP76_RS01650 | rhomboid-like protein | 3.387 | 3.413 |
| BJP76_RS02430 | histidine phosphatase family protein | 2.384 | 3.093 |
| BJP76_RS03240 | hypothetical protein | 3.825 | 3.712 |
| BJP76_RS04695 | DUF4873 domain-containing protein | 4.396 | 2.076 |
| BJP76_RS04875 | SAF domain-containing protein | 2.615 | 3.080 |
| BJP76_RS05340 | PPE family protein | 5.903 | 5.597 |
| BJP76_RS05345 | PE domain-containing protein | 6.324 | 6.455 |
| BJP76_RS05375 | YncE family protein | 2.485 | 3.318 |
| BJP76_RS05740 | 5-methyltetrahydropteroyltriglutamate--homocysteine S-methyltransferase MetE | 2.240 | 3.710 |
| BJP76_RS06030 | PPE family protein | 2.798 | 2.241 |
| BJP76_RS06060 | PPE domain-containing protein | 3.710 | 4.790 |
| BJP76_RS06250 | RNA polymerase sigma factor SigE | 3.684 | 3.026 |
| BJP76_RS07685 | hypothetical protein | 3.127 | 2.916 |
| BJP76_RS08205 | acyl-ACP desaturase | 5.288 | 2.663 |
| BJP76_RS08365 | PPE family protein | 2.631 | 3.111 |
| BJP76_RS08725 | hypothetical protein | 3.306 | 3.547 |
| BJP76_RS08775 | dimethylarginine dimethylaminohydrolase family protein | 5.483 | 3.425 |
| BJP76_RS08780 | ornithine--oxo-acid transaminase RocD | 4.005 | 2.610 |
| BJP76_RS09245 | diacylglycerol kinase | 3.057 | 2.319 |
| BJP76_RS10270 | PPE family protein | 5.733 | 4.967 |
| BJP76_RS10555 | universal stress protein | 7.241 | 2.389 |
| BJP76_RS10595 | nitroreductase family protein | 7.530 | 3.465 |
| BJP76_RS10600 | STAS/SEC14 domain-containing protein | 6.927 | 2.279 |
| BJP76_RS10605 | hypothetical protein | 8.521 | 3.693 |
| BJP76_RS10610 | nitroreductase family protein | 6.961 | 3.054 |
| BJP76_RS10615 | universal stress protein | 9.788 | 5.602 |
| BJP76_RS10620 | universal stress protein | 8.678 | 4.389 |
| BJP76_RS10625 | PAS domain S-box protein | 6.988 | 2.820 |
| BJP76_RS10680 | TetR/AcrR family transcriptional regulator | 2.220 | 2.318 |
| BJP76_RS11430 | hydrogen peroxide-inducible genes activator | 8.148 | 3.654 |
| BJP76_RS11800 | PPE family protein | 3.164 | 7.828 |
| BJP76_RS11805 | PPE family protein | 3.376 | 8.940 |
| BJP76_RS12375 | DUF4383 domain-containing protein | 8.819 | 3.949 |
| BJP76_RS13125 | ATP-binding cassette domain-containing protein | 2.627 | 2.418 |
| BJP76_RS13220 | thiol reductant ABC exporter subunit CydD | 5.352 | 2.022 |
| BJP76_RS13530 | group 1 truncated hemoglobin | 4.035 | 7.426 |
| BJP76_RS14710 | carboxylic acid reductase | 3.267 | 3.172 |
| BJP76_RS15340 | sigma-70 family RNA polymerase sigma factor SigB | 3.425 | 3.459 |
| BJP76_RS15390 | LysM peptidoglycan-binding domain-containing protein | 4.228 | 2.494 |
| BJP76_RS16220 | ammonium transporter | 2.913 | 3.105 |
| BJP76_RS17845 | group III truncated hemoglobin | 9.026 | 3.928 |
| BJP76_RS17850 | universal stress protein | 8.997 | 3.839 |
| BJP76_RS17855 | cbb3-type cytochrome c oxidase subunit I | 4.676 | 2.498 |
| BJP76_RS18340 | pyridoxamine 5'-phosphate oxidase family protein | 9.116 | 6.019 |
| BJP76_RS18345 | nitroreductase family protein | 6.046 | 3.424 |
| BJP76_RS19685 | WhiB family transcriptional regulator | 4.682 | 3.831 |
| BJP76_RS20165 | class I SAM-dependent methyltransferase | 3.452 | 3.129 |
| BJP76_RS20170 | class I SAM-dependent methyltransferase | 2.611 | 2.578 |
| BJP76_RS20760 | class I SAM-dependent methyltransferase | 3.686 | 2.369 |
| BJP76_RS20765 | class I SAM-dependent methyltransferase | 3.640 | 2.348 |
| BJP76_RS22120 | UDP-glucose/GDP-mannose dehydrogenase family protein | 4.917 | 3.928 |
| BJP76_RS22245 | DUF732 domain-containing protein | 4.471 | 3.473 |
| BJP76_RS22250 | DUF732 domain-containing protein | 2.570 | 3.018 |
| BJP76_RS22405 | PE family protein | 2.573 | 2.084 |
| BJP76_RS22415 | type VII secretion protein EccB | 3.455 | 2.304 |
| BJP76_RS22420 | type VII secretion system ESX-3 AAA family ATPase EccA3 | 3.019 | 2.331 |
| BJP76_RS22425 | PPE family protein | 5.565 | 5.360 |
| BJP76_RS22460 | PPE family protein | 5.020 | 2.536 |
| BJP76_RS22555 | uroporphyrinogen-III synthase | 3.942 | 5.607 |
| BJP76_RS22560 | sirohydrochlorin chelatase | 3.075 | 5.126 |
| BJP76_RS22620 | acyl-CoA dehydrogenase | 2.752 | 3.026 |
| BJP76_RS23305 | class I SAM-dependent methyltransferase | 2.674 | 2.309 |
| BJP76_RS23310 | class I SAM-dependent methyltransferase | 3.940 | 3.081 |
| BJP76_RS23465 | MarR family transcriptional regulator | 3.601 | 2.637 |
| BJP76_RS23470 | ATP-binding cassette domain-containing protein | 2.935 | 2.471 |
| BJP76_RS23475 | ABC transporter permease | 2.941 | 2.577 |

TABLE S6: *M. avium* genes with decreased expression in both M63 and DTT in vitro biofilm models

| **Symbol** | **Description** | **DTT log2FC** | **M63 log2FC** |
| --- | --- | --- | --- |
| BJP76_RS00670 | hypothetical protein | -3.079 | -2.558 |
| BJP76_RS01260 | lipoprotein LpqH | -2.011 | -3.468 |
| BJP76_RS02025 | DUF4193 domain-containing protein | -3.533 | -2.350 |
| BJP76_RS04115 | cytochrome c oxidase subunit 3 family protein | -2.312 | -2.073 |
| BJP76_RS06650 | DUF732 domain-containing protein | -2.113 | -4.430 |
| BJP76_RS10845 | MPT63 family protein | -3.173 | -2.599 |
| BJP76_RS11345 | transglycosylase family protein | -4.828 | -2.237 |
| BJP76_RS13680 | putative sugar O-methyltransferase | -2.963 | -2.330 |
| BJP76_RS14140 | DUF732 domain-containing protein | -3.849 | -2.856 |
| BJP76_RS14145 | PPE family protein | -2.917 | -2.297 |
| BJP76_RS16030 | MPT63 family protein | -3.585 | -2.972 |
| BJP76_RS18205 | glycosyltransferase family 2 protein | -4.228 | -2.421 |
| BJP76_RS20900 | SulP family inorganic anion transporter | -2.038 | -4.262 |
| BJP76_RS21715 | hypothetical protein | -2.009 | -2.515 |

TABLE S7: Top 30 genes with greatest expression increase and bottom 30 genes with greatest expression decrease in planktonic *M. avium* post macrophage infection

| **Symbol** | **Description** | **log2FC** | **logCPM** | **PValue** | **FDR** |
| --- | --- | --- | --- | --- | --- |
| BJP76_RS11805 | PPE family protein | 9.260 | 8.750 | 4.61E-109 | 9.86E-106 |
| BJP76_RS05345 | PE domain-containing protein | 8.977 | 9.692 | 6.81E-52 | 2.91E-49 |
| BJP76_RS21420 | isocitrate lyase AceA | 8.699 | 11.687 | 2.88E-38 | 5.37E-36 |
| BJP76_RS05340 | PPE family protein | 8.646 | 9.977 | 3.24E-49 | 1.15E-46 |
| BJP76_RS11800 | PPE family protein | 8.556 | 8.411 | 1.24E-77 | 1.06E-74 |
| BJP76_RS08730 | bifunctional nitrate reductase/sulfite reductase flavoprotein subunit alpha | 8.419 | 9.760 | 1.12E-139 | 4.77E-136 |
| BJP76_RS13530 | group 1 truncated hemoglobin | 8.242 | 6.955 | 1.39E-32 | 1.61E-30 |
| BJP76_RS19645 | TauD/TfdA family dioxygenase | 7.968 | 11.439 | 5.59E-79 | 5.98E-76 |
| BJP76_RS05120 | dTDP-4-dehydrorhamnose 3,5-epimerase family protein | 7.803 | 6.538 | 7.66E-75 | 5.47E-72 |
| BJP76_RS14710 | carboxylic acid reductase | 7.405 | 12.107 | 4.75E-38 | 8.47E-36 |
| BJP76_RS11795 | DUF732 domain-containing protein | 7.316 | 7.052 | 9.83E-42 | 2.21E-39 |
| BJP76_RS22580 | Hsp20/alpha crystallin family protein | 6.968 | 8.911 | 9.46E-32 | 1.04E-29 |
| BJP76_RS05125 | PPE family protein | 6.609 | 8.231 | 2.96E-29 | 2.59E-27 |
| BJP76_RS22525 | nitrate/nitrite transporter | 6.527 | 9.716 | 4.92E-47 | 1.62E-44 |
| BJP76_RS13930 | thioredoxin TrxA | 6.441 | 9.985 | 7.21E-31 | 7.18E-29 |
| BJP76_RS22555 | uroporphyrinogen-III synthase | 6.370 | 7.243 | 1.83E-53 | 8.71E-51 |
| BJP76_RS06475 | LLM class flavin-dependent oxidoreductase | 6.179 | 9.435 | 4.47E-26 | 2.94E-24 |
| BJP76_RS22550 | nitrate/nitrite transporter | 6.035 | 7.795 | 7.40E-42 | 1.76E-39 |
| BJP76_RS10915 | helix-turn-helix transcriptional regulator | 6.012 | 7.120 | 5.11E-12 | 5.79E-11 |
| BJP76_RS23470 | ATP-binding cassette domain-containing protein | 5.985 | 8.111 | 5.02E-30 | 4.67E-28 |
| BJP76_RS19850 | hypothetical protein | 5.948 | 3.256 | 4.52E-41 | 9.68E-39 |
| BJP76_RS05115 | sugar transferase | 5.891 | 7.918 | 1.71E-50 | 6.67E-48 |
| BJP76_RS14855 | ferredoxin family protein | 5.872 | 8.357 | 5.73E-43 | 1.53E-40 |
| BJP76_RS11790 | DUF732 domain-containing protein | 5.868 | 5.572 | 9.75E-63 | 5.22E-60 |
| BJP76_RS22560 | sirohydrochlorin chelatase | 5.847 | 7.028 | 7.13E-36 | 1.02E-33 |
| BJP76_RS10035 | MCE family protein | -3.281 | 3.998 | 5.61E-15 | 1.05E-13 |
| BJP76_RS07420 | ferredoxin | -3.292 | 2.880 | 1.38E-10 | 1.23E-09 |
| BJP76_RS00440 | FAD-binding oxidoreductase | -3.319 | 7.371 | 1.94E-16 | 4.64E-15 |
| BJP76_RS18235 | PfkB family carbohydrate kinase | -3.359 | 2.238 | 3.06E-08 | 1.82E-07 |
| BJP76_RS22310 | Hsp70 family protein | -3.388 | 7.127 | 2.76E-21 | 1.18E-19 |
| BJP76_RS01395 | cellulase family glycosylhydrolase | -3.433 | 7.211 | 4.88E-24 | 2.61E-22 |
| BJP76_RS18145 | Rho termination factor N-terminal domain-containing protein | -3.471 | 4.733 | 1.52E-06 | 6.48E-06 |
| BJP76_RS10505 | DUF3349 domain-containing protein | -3.478 | 7.190 | 1.86E-15 | 3.71E-14 |
| BJP76_RS08460 | hypothetical protein | -3.498 | 8.511 | 1.55E-21 | 6.78E-20 |
| BJP76_RS12865 | hypothetical protein | -3.668 | 6.455 | 6.46E-16 | 1.41E-14 |
| BJP76_RS07685 | hypothetical protein | -3.697 | 8.506 | 1.13E-05 | 4.12E-05 |
| BJP76_RS18205 | glycosyltransferase family 2 protein | -3.759 | 2.030 | 1.41E-05 | 5.03E-05 |
| BJP76_RS11940 | ANTAR domain-containing protein | -3.784 | 7.046 | 1.25E-26 | 9.06E-25 |
| BJP76_RS16265 | cell division protein SepIVA | -3.834 | 10.431 | 3.59E-35 | 4.81E-33 |
| BJP76_RS21720 | hypothetical protein | -3.853 | 5.725 | 2.07E-25 | 1.32E-23 |
| BJP76_RS18105 | alpha/beta hydrolase | -3.860 | 6.732 | 3.67E-16 | 8.45E-15 |
| BJP76_RS18220 | HAD-IIIA family hydrolase | -3.862 | 0.881 | 2.14E-06 | 8.88E-06 |
| BJP76_RS13895 | NlpC/P60 family peptidoglycan endopeptidase RipA | -3.867 | 8.612 | 1.04E-24 | 5.84E-23 |
| BJP76_RS00195 | WhiB family transcriptional regulator | -3.943 | 6.210 | 5.22E-08 | 2.99E-07 |
| BJP76_RS22240 | hypothetical protein | -3.950 | 6.330 | 1.70E-18 | 5.27E-17 |
| BJP76_RS18905 | NnrU family protein | -3.952 | 6.712 | 1.49E-22 | 7.06E-21 |
| BJP76_RS23510 | elongation factor G-like protein EF-G2 | -3.972 | 9.077 | 1.20E-38 | 2.33E-36 |
| BJP76_RS01260 | lipoprotein LpqH | -4.059 | 7.665 | 8.41E-25 | 5.00E-23 |
| BJP76_RS22005 | hypothetical protein | -4.073 | 2.587 | 1.43E-10 | 1.26E-09 |
| BJP76_RS02170 | hypothetical protein | -4.259 | 7.583 | 5.93E-28 | 4.98E-26 |
| BJP76_RS02515 | 2-polyprenyl-6-methoxyphenol hydroxylase | -4.347 | 9.652 | 2.70E-09 | 1.93E-08 |
| BJP76_RS13890 | NlpC/P60 family peptidoglycan endopeptidase RipB | -4.388 | 7.449 | 1.83E-34 | 2.31E-32 |
| BJP76_RS06650 | DUF732 domain-containing protein | -4.755 | 7.061 | 1.22E-18 | 3.88E-17 |
| BJP76_RS11345 | transglycosylase family protein | -4.927 | 5.854 | 1.58E-37 | 2.40E-35 |
| BJP76_RS13195 | hypothetical protein | -5.865 | 7.134 | 2.60E-21 | 1.12E-19 |

TABLE S8: Top go terms enriched in planktonic *M. avium* post macrophage infection

|  | **GO.ID** | **Term** | **Annotated genes** | **Significant DE genes (FDR <0.05)** | **Expected DE genes** | **Weighted Fisher-exact p-value** |
| --- | --- | --- | --- | --- | --- | --- |
| Biological process (BP) | GO:0045892 | negative regulation of DNA-templated transcription | 10 | 10 | 6.2 | 0.008 |
|  | GO:0032787 | monocarboxylic acid metabolic process | 113 | 63 | 69.8 | 0.019 |
|  | GO:0006541 | glutamine metabolic process* | 21 | 17 | 13.0 | 0.051 |
|  | GO:0019752 | carboxylic acid metabolic process* | 280 | 182 | 172.9 | 0.053 |
|  | GO:0008360 | regulation of cell shape* | 20 | 16 | 12.4 | 0.068 |
|  | GO:0009236 | cobalamin biosynthetic process | 13 | 11 | 8.0 | 0.073 |
|  | GO:0006091 | generation of precursor metabolites and energy* | 75 | 50 | 46.3 | 0.085 |
|  | GO:0006163 | purine nucleotide metabolic process | 64 | 40 | 39.5 | 0.086 |
|  | GO:0043933 | protein-containing complex organization | 17 | 12 | 10.5 | 0.090 |
|  | GO:0044255 | cellular lipid metabolic process | 115 | 67 | 71.0 | 0.092 |
| Cellular component (CC) | GO:0000428 | DNA-directed RNA polymerase complex | 17 | 15 | 10.5 | 0.018 |
|  | GO:0005829 | cytosol | 34 | 26 | 21.1 | 0.054 |
| Molecular function (MF) | GO:0044183 | protein folding chaperone | 10 | 10 | 6.1 | 0.008 |
|  | GO:0050661 | NADP binding | 37 | 30 | 22.7 | 0.008 |
|  | GO:0070566 | adenylyltransferase activity | 16 | 14 | 9.8 | 0.024 |
|  | GO:0008408 | 3'-5' exonuclease activity | 10 | 9 | 6.1 | 0.055 |
|  | GO:0008483 | transaminase activity^#^ | 27 | 21 | 16.6 | 0.056 |
|  | GO:0008237 | metallopeptidase activity | 27 | 21 | 16.6 | 0.058 |
|  | GO:0016705 | oxidoreductase activity, acting on paired donors, with incorporation or reduction of molecular oxygen* | 146 | 99 | 89.7 | 0.060 |
|  | GO:0004519 | endonuclease activity | 50 | 36 | 30.7 | 0.078 |
|  | GO:0016773 | phosphotransferase activity, alcohol group as acceptor* | 65 | 40 | 39.9 | 0.081 |
|  | GO:0042578 | phosphoric ester hydrolase activity | 35 | 24 | 21.5 | 0.082 |
|  | GO:0004601 | peroxidase activity^#^ | 35 | 26 | 21.5 | 0.086 |
|  | GO:0046873 | metal ion transmembrane transporter activity | 22 | 17 | 13.5 | 0.092 |
|  | GO:0016638 | oxidoreductase activity, acting on the CH-NH2 group of donors | 12 | 10 | 7.4 | 0.099 |
|  | GO:0004016 | adenylate cyclase activity | 12 | 10 | 7.4 | 0.099 |
|  |  | *same or closely related term enriched in M63-derived *M. avium* post infection | | | | |
|  |  | #enriched in M63 biofilm prior to infection | | | | |

TABLE S9: Top 30 genes with greatest expression increase and bottom 30 genes with greatest expression decrease in M63 biofilm *M. avium* post macrophage infection

| **Symbol** | **Description** | **log2FC** | **logCPM** | **PValue** | **FDR** |
| --- | --- | --- | --- | --- | --- |
| BJP76_RS19645 | TauD/TfdA family dioxygenase | 7.084 | 11.439 | 1.97E-41 | 8.43E-38 |
| BJP76_RS21420 | isocitrate lyase AceA | 7.008 | 11.687 | 4.84E-18 | 2.96E-15 |
| BJP76_RS14855 | ferredoxin family protein | 6.450 | 8.357 | 4.48E-30 | 6.40E-27 |
| BJP76_RS14850 | helix-turn-helix domain-containing protein | 5.575 | 8.470 | 2.98E-26 | 3.19E-23 |
| BJP76_RS14365 | ferredoxin family protein | 5.270 | 2.142 | 6.97E-07 | 1.95E-05 |
| BJP76_RS18830 | thiolase family protein | 5.225 | 8.405 | 1.07E-13 | 3.07E-11 |
| BJP76_RS08675 | ABC transporter permease | 5.197 | 5.422 | 2.77E-08 | 1.37E-06 |
| BJP76_RS08690 | MCE family protein | 5.104 | 5.647 | 2.64E-08 | 1.36E-06 |
| BJP76_RS08670 | ABC transporter permease | 5.002 | 6.440 | 4.86E-08 | 2.17E-06 |
| BJP76_RS14860 | class I SAM-dependent methyltransferase | 4.939 | 7.925 | 1.42E-19 | 1.02E-16 |
| BJP76_RS08845 | antitoxin MazE family protein | 4.891 | -0.473 | 6.62E-09 | 3.99E-07 |
| BJP76_RS08710 | hypothetical protein | 4.792 | 5.276 | 1.68E-07 | 5.78E-06 |
| BJP76_RS14845 | hypothetical protein | 4.737 | 6.288 | 1.56E-20 | 1.34E-17 |
| BJP76_RS08680 | MCE family protein | 4.679 | 5.715 | 3.10E-10 | 3.16E-08 |
| BJP76_RS14710 | carboxylic acid reductase | 4.636 | 12.107 | 1.40E-12 | 2.72E-10 |
| BJP76_RS08695 | MCE family protein | 4.635 | 5.262 | 8.04E-08 | 3.19E-06 |
| BJP76_RS08685 | MCE family protein | 4.609 | 5.031 | 9.98E-07 | 2.61E-05 |
| BJP76_RS18835 | DUF302 domain-containing protein | 4.452 | 5.329 | 1.42E-09 | 1.19E-07 |
| BJP76_RS21390 | helix-turn-helix transcriptional regulator | 4.397 | 7.893 | 2.24E-13 | 5.06E-11 |
| BJP76_RS08250 | fumarylacetoacetate hydrolase family protein | 4.376 | 3.880 | 8.31E-07 | 2.25E-05 |
| BJP76_RS10925 | J domain-containing protein | 4.362 | 6.957 | 3.75E-05 | 5.03E-04 |
| BJP76_RS18840 | metal-dependent hydrolase | 4.331 | 3.423 | 4.03E-10 | 3.92E-08 |
| BJP76_RS08715 | membrane protein | 4.324 | 4.519 | 5.95E-09 | 3.69E-07 |
| BJP76_RS08245 | VOC family protein | 4.299 | 4.708 | 5.65E-06 | 1.05E-04 |
| BJP76_RS20940 | glycosyltransferase family 1 protein | 4.282 | 7.751 | 1.08E-16 | 5.13E-14 |
| BJP76_RS11460 | endopeptidase La | 4.272 | 10.277 | 1.80E-09 | 1.45E-07 |
| BJP76_RS23320 | pirin family protein | 4.188 | 7.438 | 2.07E-10 | 2.27E-08 |
| BJP76_RS14475 | luciferase | 4.107 | 5.251 | 5.39E-08 | 2.30E-06 |
| BJP76_RS18320 | HSP20 family small heat-shock protein | 4.071 | 6.167 | 6.61E-06 | 1.20E-04 |
| BJP76_RS13930 | thioredoxin TrxA | 4.061 | 9.985 | 2.93E-10 | 3.06E-08 |
| BJP76_RS13570 | hemerythrin domain-containing protein | -2.220 | 7.244 | 9.77E-05 | 1.11E-03 |
| BJP76_RS14890 | hemerythrin domain-containing protein | -2.221 | 6.368 | 6.52E-04 | 5.15E-03 |
| BJP76_RS06075 | proline dehydrogenase family protein | -2.224 | 8.102 | 1.41E-03 | 9.85E-03 |
| BJP76_RS17865 | slipin family protein | -2.260 | 8.260 | 1.36E-04 | 1.46E-03 |
| BJP76_RS19090 | phosphate ABC transporter substrate-binding protein PstS | -2.276 | 8.456 | 2.18E-04 | 2.15E-03 |
| BJP76_RS00700 | acyl-CoA dehydrogenase family protein | -2.304 | 5.289 | 9.50E-10 | 8.40E-08 |
| BJP76_RS05945 | nitrate reductase subunit beta NarH | -2.309 | 7.104 | 3.25E-08 | 1.56E-06 |
| BJP76_RS22405 | acyl-CoA desaturase | -2.310 | 4.844 | 7.34E-07 | 2.01E-05 |
| BJP76_RS21630 | hypothetical protein | -2.328 | 8.278 | 1.90E-10 | 2.20E-08 |
| BJP76_RS10045 | TetR family transcriptional regulator | -2.342 | 4.605 | 1.54E-04 | 1.63E-03 |
| BJP76_RS18860 | DUF3263 domain-containing protein | -2.343 | 7.111 | 9.06E-05 | 1.04E-03 |
| BJP76_RS10510 | hypothetical protein | -2.431 | 4.201 | 1.92E-06 | 4.46E-05 |
| BJP76_RS23395 | cell division protein SepIVA | -2.459 | 8.365 | 1.31E-05 | 2.05E-04 |
| BJP76_RS05955 | respiratory nitrate reductase subunit gamma | -2.462 | 5.854 | 6.31E-11 | 9.65E-09 |
| BJP76_RS03695 | hypothetical protein | -2.522 | 6.608 | 9.09E-04 | 6.85E-03 |
| BJP76_RS17855 | slipin family protein | -2.531 | 9.207 | 1.04E-05 | 1.72E-04 |
| BJP76_RS15905 | PPE family protein | -2.534 | 4.286 | 1.92E-05 | 2.85E-04 |
| BJP76_RS00265 | LpqN/LpqT family lipoprotein | -2.591 | 6.966 | 1.30E-09 | 1.11E-07 |
| BJP76_RS13890 | Rieske 2Fe-2S domain-containing protein | -2.603 | 7.449 | 2.06E-10 | 2.27E-08 |
| BJP76_RS01240 | Rv3717 family N-acetylmuramoyl-L-alanine amidase | -2.726 | 6.407 | 3.79E-04 | 3.39E-03 |
| BJP76_RS10050 | PPE family protein | -2.732 | 5.832 | 1.10E-05 | 1.79E-04 |
| BJP76_RS10825 | cellulose-binding domain-containing protein | -2.760 | 6.734 | 1.73E-06 | 4.09E-05 |
| BJP76_RS14895 | Rieske 2Fe-2S domain-containing protein | -2.803 | 8.363 | 5.90E-08 | 2.40E-06 |
| BJP76_RS11940 | hypothetical protein | -2.803 | 7.046 | 2.19E-11 | 4.08E-09 |
| BJP76_RS22245 | PE family protein | -2.914 | 9.848 | 7.46E-06 | 1.32E-04 |
| BJP76_RS02515 | helix-turn-helix domain-containing protein | -2.919 | 9.652 | 1.03E-03 | 7.57E-03 |
| BJP76_RS22240 | DUF732 domain-containing protein | -2.939 | 6.330 | 1.44E-07 | 5.09E-06 |
| BJP76_RS15910 | cbb3-type cytochrome c oxidase subunit I | -3.076 | 7.014 | 3.48E-10 | 3.47E-08 |
| BJP76_RS13195 | Rv1535 family protein | -3.123 | 7.134 | 2.76E-05 | 3.89E-04 |
| BJP76_RS07685 | YggT family protein | -7.029 | 8.506 | 5.05E-08 | 2.23E-06 |

TABLE S10: Top go terms enriched in M63 biofilm *M. avium*  post macrophage infection (relative to M63 biofilm *M. avium*)

|  | **GO.ID** | **Term** | **Annotated genes** | **Significant DE genes (FDR <0.05)** | **Expected DE genes** | **Weighted Fisher-exact p-value** |
| --- | --- | --- | --- | --- | --- | --- |
| Biological process (BP) | GO:0009064 | glutamine family amino acid metabolic process* | 35 | 14 | 11.2 | 0.005 |
|  | GO:0006807 | nitrogen compound metabolic process | 981 | 313 | 313.4 | 0.006 |
|  | GO:0006091 | generation of precursor metabolites and energy* | 75 | 30 | 24.0 | 0.006 |
|  | GO:0008360 | regulation of cell shape* | 20 | 12 | 6.4 | 0.009 |
|  | GO:0005975 | carbohydrate metabolic process | 105 | 49 | 33.6 | 0.010 |
|  | GO:1901606 | alpha-amino acid catabolic process | 12 | 8 | 3.8 | 0.014 |
|  | GO:0009063 | amino acid catabolic process | 12 | 8 | 3.8 | 0.014 |
|  | GO:0006534 | cysteine metabolic process | 13 | 8 | 4.2 | 0.026 |
|  | GO:0016042 | lipid catabolic process | 23 | 12 | 7.4 | 0.034 |
|  | GO:0043648 | dicarboxylic acid metabolic process* | 41 | 14 | 13.1 | 0.035 |
|  | GO:0046483 | heterocycle metabolic process | 647 | 199 | 206.7 | 0.035 |
|  | GO:1901360 | organic cyclic compound metabolic process | 684 | 211 | 218.5 | 0.035 |
|  | GO:0006163 | purine nucleotide metabolic process | 64 | 20 | 20.5 | 0.035 |
|  | GO:0009082 | branched-chain amino acid biosynthetic process | 19 | 9 | 6.1 | 0.037 |
|  | GO:0009252 | peptidoglycan biosynthetic process | 22 | 11 | 7.0 | 0.059 |
|  | GO:0055082 | intracellular chemical homeostasis | 10 | 6 | 3.2 | 0.063 |
|  | GO:0006629 | lipid metabolic process | 170 | 68 | 54.3 | 0.068 |
|  | GO:1901135 | carbohydrate derivative metabolic process | 151 | 61 | 48.2 | 0.072 |
|  | GO:0044238 | primary metabolic process | 1113 | 374 | 355.6 | 0.080 |
|  | GO:0032787 | monocarboxylic acid metabolic process | 113 | 42 | 36.1 | 0.082 |
|  | GO:0065008 | regulation of biological quality | 26 | 16 | 8.3 | 0.084 |
|  | GO:0005996 | monosaccharide metabolic process | 16 | 9 | 5.1 | 0.098 |
|  | GO:0051707 | response to other organism | 16 | 5 | 5.1 | 0.100 |
| Cellular component (CC) | GO:0005829 | cytosol | 34 | 15 | 10.8 | 0.087 |
|  | GO:0016020 | membrane | 1042 | 342 | 331.6 | 0.090 |
| Molecular function (MF) | GO:0003995 | acyl-CoA dehydrogenase activity | 15 | 10 | 5.0 | 0.008 |
|  | GO:0016705 | oxidoreductase activity, acting on paired donors, with incorporation or reduction of molecular oxygen* | 146 | 62 | 48.6 | 0.011 |
|  | GO:0050660 | flavin adenine dinucleotide binding | 115 | 48 | 38.3 | 0.013 |
|  | GO:0000287 | magnesium ion binding | 80 | 36 | 26.7 | 0.018 |
|  | GO:0042626 | ATPase-coupled transmembrane transporter activity | 42 | 11 | 14.0 | 0.020 |
|  | GO:0030976 | thiamine pyrophosphate binding | 15 | 9 | 5.0 | 0.030 |
|  | GO:0016831 | carboxy-lyase activity | 56 | 26 | 18.7 | 0.032 |
|  | GO:0051537 | 2 iron, 2 sulfur cluster binding | 33 | 16 | 11.0 | 0.050 |
|  | GO:0016833 | oxo-acid-lyase activity | 12 | 7 | 4.0 | 0.066 |
|  | GO:0016462 | pyrophosphatase activity | 146 | 56 | 48.6 | 0.074 |
|  | GO:0031177 | phosphopantetheine binding | 10 | 6 | 3.3 | 0.076 |
|  | GO:0016773 | phosphotransferase activity, alcohol group as acceptor* | 65 | 23 | 21.7 | 0.087 |
|  | GO:0022853 | active monoatomic ion transmembrane tran... | 20 | 7 | 6.7 | 0.088 |
|  | GO:0016836 | hydro-lyase activity | 70 | 29 | 23.3 | 0.092 |
|  | GO:0016874 | ligase activity | 157 | 51 | 52.3 | 0.093 |
|  |  | *same or closely related term enriched in M63-derived *M. avium* post infection | | | | |

TABLE S11: *M. avium* genes with increased expression in M63 biofilm (pre infection), planktonic culture post infection, no change in expression in M63 biofilm cells post infection

| **Symbol** | **Description** | **M63 log2FC** | **Plank post-inf log2FC** | **M63 post-inf log2FC** |
| --- | --- | --- | --- | --- |
| BJP76_RS00395 | class I SAM-dependent methyltransferase | 2.066 | 2.5 | 1.355 |
| BJP76_RS00400 | TetR family transcriptional regulator | 2.267 | 3.721 | 1.47 |
| BJP76_RS00410 | MmpS family transport accessory protein | 3.191 | 4.063 | 1.154 |
| BJP76_RS00415 | RND family transporter | 2.646 | 4.362 | 1.915 |
| BJP76_RS00460 | LysR family transcriptional regulator | 2.019 | 3.466 | 1.543 |
| BJP76_RS00825 | DNA polymerase IV DinB | 2.02 | 2.189 | 0.901 |
| BJP76_RS01245 | two-component system response regulator TcrX | 2.41 | 2.628 | -0.571 |
| BJP76_RS01650 | rhomboid-like protein | 3.413 | 5.105 | 1.3 |
| BJP76_RS02185 | TadA family conjugal transfer-associated ATPase | 2.772 | 2.062 | 0.857 |
| BJP76_RS02385 | DUF2520 domain-containing protein | 2.87 | 2.127 | 0.303 |
| BJP76_RS03240 | hypothetical protein | 3.712 | 2.047 | -0.909 |
| BJP76_RS05125 | PPE family protein | 4.313 | 6.609 | 1.546 |
| BJP76_RS05375 | YncE family protein | 3.318 | 2.348 | -0.812 |
| BJP76_RS05410 | cysteine dioxygenase | 2.115 | 2.834 | 0.963 |
| BJP76_RS05940 | nitrate reductase subunit alpha | 3.828 | 2.431 | -1.905 |
| BJP76_RS05950 | nitrate reductase molybdenum cofactor assembly chaperone NarJ | 3.314 | 2.076 | -1.846 |
| BJP76_RS06060 | PPE domain-containing protein | 4.79 | 3.068 | -0.438 |
| BJP76_RS06250 | RNA polymerase sigma factor SigE | 3.026 | 2.437 | -0.105 |
| BJP76_RS06605 | adenylyl-sulfate kinase CysC | 2.334 | 3.002 | 0.378 |
| BJP76_RS07080 | pyridoxamine 5'-phosphate oxidase family protein | 3.266 | 4.038 | 1.991 |
| BJP76_RS07955 | isocitrate lyase/PEP mutase family protein | 3.36 | 2.485 | 0.755 |
| BJP76_RS08195 | sodium-dependent bicarbonate transport family permease | 3.32 | 4.036 | -0.411 |
| BJP76_RS08365 | PPE family protein | 3.111 | 3.378 | 1.152 |
| BJP76_RS08725 | hypothetical protein | 3.547 | 4.231 | 0.372 |
| BJP76_RS08730 | bifunctional nitrate reductase/sulfite reductase flavoprotein subunit alpha | 6.823 | 8.419 | 1.26 |
| BJP76_RS08775 | dimethylarginine dimethylaminohydrolase family protein | 3.425 | 3.535 | 0.028 |
| BJP76_RS08780 | ornithine--oxo-acid transaminase RocD | 2.61 | 2.954 | -0.373 |
| BJP76_RS09070 | type II toxin-antitoxin system HicB family antitoxin | 3.92 | 2.003 | 0.012 |
| BJP76_RS09085 | sirohydrochlorin chelatase | 3.232 | 2.634 | 0.228 |
| BJP76_RS09090 | phosphoadenylyl-sulfate reductase | 3.251 | 3.127 | 0.656 |
| BJP76_RS09095 | nitrite/sulfite reductase | 3.442 | 3.834 | 1.136 |
| BJP76_RS09430 | 3-methyl-2-oxobutanoate hydroxymethyltransferase PanB | 3.932 | 2.801 | -0.702 |
| BJP76_RS10555 | universal stress protein | 2.389 | 3.75 | 0.695 |
| BJP76_RS10605 | hypothetical protein | 3.693 | 2.458 | -1.452 |
| BJP76_RS10610 | nitroreductase family protein | 3.054 | 2.788 | -0.788 |
| BJP76_RS10615 | universal stress protein | 5.602 | 5.841 | -0.697 |
| BJP76_RS10620 | universal stress protein | 4.389 | 4.271 | -0.884 |
| BJP76_RS10625 | PAS domain S-box protein | 2.82 | 3.172 | -0.489 |
| BJP76_RS11430 | hydrogen peroxide-inducible genes activator | 3.654 | 5.468 | 1.467 |
| BJP76_RS11790 | DUF732 domain-containing protein | 5.943 | 5.868 | -0.029 |
| BJP76_RS11795 | DUF732 domain-containing protein | 6.779 | 7.316 | 0.647 |
| BJP76_RS11800 | PPE family protein | 7.828 | 8.556 | 0.808 |
| BJP76_RS11805 | PPE family protein | 8.94 | 9.26 | 0.568 |
| BJP76_RS11810 | DUF732 domain-containing protein | 2.267 | 3.345 | 1.072 |
| BJP76_RS11820 | PPE family protein | 3.339 | 4.419 | 0.316 |
| BJP76_RS11825 | PE family protein | 4.253 | 4.326 | -0.623 |
| BJP76_RS12000 | class I SAM-dependent methyltransferase | 3.172 | 2.888 | -0.504 |
| BJP76_RS12375 | DUF4383 domain-containing protein | 3.949 | 3.372 | -1.721 |
| BJP76_RS13530 | group 1 truncated hemoglobin | 7.426 | 8.242 | 1.01 |
| BJP76_RS13690 | glycosyltransferase | 2.195 | 2.14 | -0.463 |
| BJP76_RS13980 | metalloregulator ArsR/SmtB family transcription factor | 2.435 | 3.89 | 1.315 |
| BJP76_RS14240 | methyltransferase domain-containing protein | 6.96 | 5.57 | -1.038 |
| BJP76_RS15340 | sigma-70 family RNA polymerase sigma factor SigB | 3.459 | 2.556 | -0.672 |
| BJP76_RS15390 | LysM peptidoglycan-binding domain-containing protein | 2.494 | 2.811 | 0.411 |
| BJP76_RS15500 | transcriptional regulator ClgR | 2.422 | 2.108 | 0.288 |
| BJP76_RS16325 | FtsK/SpoIIIE domain-containing protein | 2.368 | 2.326 | -0.931 |
| BJP76_RS16485 | transglycosylase SLT domain-containing protein | 2.266 | 3.766 | 1.461 |
| BJP76_RS16500 | SDR family NAD(P)-dependent oxidoreductase | 2.211 | 2.739 | 0.88 |
| BJP76_RS17845 | group III truncated hemoglobin | 3.928 | 4.94 | 0.244 |
| BJP76_RS17850 | universal stress protein | 3.839 | 5.414 | 0.852 |
| BJP76_RS18340 | pyridoxamine 5'-phosphate oxidase family protein | 6.019 | 4.553 | -1.461 |
| BJP76_RS18855 | TetR/AcrR family transcriptional regulator | 3.727 | 2.722 | 0.436 |
| BJP76_RS18915 | hypothetical protein | 2.985 | 2.941 | 0.215 |
| BJP76_RS19685 | WhiB family transcriptional regulator | 3.831 | 5.762 | 1.658 |
| BJP76_RS20090 | ABC transporter permease | 3.068 | 2.218 | 0.69 |
| BJP76_RS20135 | class I SAM-dependent methyltransferase | 2.561 | 2.657 | -0.465 |
| BJP76_RS20165 | class I SAM-dependent methyltransferase | 3.129 | 2.597 | 1.25 |
| BJP76_RS20170 | class I SAM-dependent methyltransferase | 2.578 | 2.211 | 1.182 |
| BJP76_RS20375 | aldo/keto reductase | 3.9 | 2.008 | -1.54 |
| BJP76_RS20760 | class I SAM-dependent methyltransferase | 2.369 | 2.776 | -0.146 |
| BJP76_RS20765 | class I SAM-dependent methyltransferase | 2.348 | 2.777 | 0.105 |
| BJP76_RS22070 | sugar porter family MFS transporter | 2.776 | 2.68 | -0.297 |
| BJP76_RS22120 | UDP-glucose/GDP-mannose dehydrogenase family protein | 3.928 | 5.193 | 0.259 |
| BJP76_RS22425 | PPE family protein | 5.36 | 4.52 | -0.443 |
| BJP76_RS22460 | PPE family protein | 2.536 | 3.182 | 1.5 |
| BJP76_RS22550 | nitrate/nitrite transporter | 6.13 | 6.035 | -1.121 |
| BJP76_RS22555 | uroporphyrinogen-III synthase | 5.607 | 6.37 | 0.359 |
| BJP76_RS22560 | sirohydrochlorin chelatase | 5.126 | 5.847 | 0.374 |
| BJP76_RS22565 | hypothetical protein | 3.958 | 3.823 | -0.253 |
| BJP76_RS22570 | nitrite reductase small subunit NirD | 4.183 | 4.77 | 0.363 |
| BJP76_RS22575 | nitrite reductase large subunit NirB | 4.875 | 5.629 | 0.467 |
| BJP76_RS22620 | acyl-CoA dehydrogenase | 3.026 | 2.132 | 0.335 |
| BJP76_RS22960 | TetR/AcrR family transcriptional regulator | 2.527 | 3.446 | 1.64 |
| BJP76_RS23305 | class I SAM-dependent methyltransferase | 2.309 | 2.62 | 0.895 |
| BJP76_RS23310 | class I SAM-dependent methyltransferase | 3.081 | 3.374 | 1.195 |
| BJP76_RS23455 | metal-sensitive transcriptional regulator | 2.951 | 3.31 | 1.015 |
| BJP76_RS23550 | NRAMP family divalent metal transporter | 2.93 | 2.773 | -0.799 |

TABLE S12: *M. avium* genes with decreased expression in M63 biofilm (pre infection), planktonic culture post infection, no change in expression in M63 biofilm cells post infection

| **Symbol** | **Description** | **M63 log2FC** | **Plank post-inf log2FC** | **M63 post-inf log2FC** |
| --- | --- | --- | --- | --- |
| BJP76_RS00155 | hypothetical protein | -2.297 | -2.015 | -0.241 |
| BJP76_RS00190 | acyl-CoA dehydrogenase family protein | -2.816 | -2.513 | -0.476 |
| BJP76_RS00195 | WhiB family transcriptional regulator | -3.864 | -3.943 | -1.096 |
| BJP76_RS00405 | hypothetical protein | -2.326 | -2.171 | -0.402 |
| BJP76_RS00670 | hypothetical protein | -2.558 | -3.281 | -0.681 |
| BJP76_RS00740 | WXG100 family type VII secretion target | -2.574 | -2.588 | -0.179 |
| BJP76_RS01260 | lipoprotein LpqH | -3.468 | -4.059 | -0.858 |
| BJP76_RS02025 | DUF4193 domain-containing protein | -2.350 | -3.040 | -0.401 |
| BJP76_RS03850 | amidohydrolase family protein | -2.092 | -2.841 | -0.984 |
| BJP76_RS03890 | ferredoxin | -2.547 | -2.497 | 0.268 |
| BJP76_RS05805 | aldehyde dehydrogenase family protein | -2.551 | -2.387 | 0.027 |
| BJP76_RS05905 | hypothetical protein | -2.472 | -2.969 | -0.522 |
| BJP76_RS06630 | tetratricopeptide repeat protein | -3.159 | -2.383 | 0.226 |
| BJP76_RS06645 | NUDIX hydrolase | -2.889 | -2.676 | 0.105 |
| BJP76_RS06650 | DUF732 domain-containing protein | -4.430 | -4.755 | -0.543 |
| BJP76_RS08325 | hypothetical protein | -2.079 | -2.202 | -0.070 |
| BJP76_RS09385 | cytochrome P450 | -2.182 | -3.118 | -0.241 |
| BJP76_RS10845 | MPT63 family protein | -2.599 | -2.952 | -0.343 |
| BJP76_RS11345 | transglycosylase family protein | -2.237 | -4.927 | -1.787 |
| BJP76_RS11770 | DUF732 domain-containing protein | -3.672 | -3.192 | 0.578 |
| BJP76_RS11775 | PPE family protein | -2.806 | -2.404 | 0.125 |
| BJP76_RS11780 | PPE family protein | -2.523 | -2.855 | -0.243 |
| BJP76_RS11970 | enoyl-CoA hydratase-related protein | -2.523 | -2.835 | -0.145 |
| BJP76_RS12865 | hypothetical protein | -2.768 | -3.668 | -1.385 |
| BJP76_RS13680 | putative sugar O-methyltransferase | -2.330 | -2.803 | -0.850 |
| BJP76_RS14130 | wax ester/triacylglycerol synthase family O-acyltransferase | -2.020 | -2.806 | -0.797 |
| BJP76_RS16030 | MPT63 family protein | -2.972 | -2.212 | -0.324 |
| BJP76_RS16560 | HU family DNA-binding protein | -2.694 | -2.992 | -0.118 |
| BJP76_RS17685 | fluoride efflux transporter CrcB | -2.791 | -2.675 | -0.148 |
| BJP76_RS18100 | flavin monoamine oxidase family protein | -3.279 | -3.115 | -0.157 |
| BJP76_RS18105 | alpha/beta hydrolase | -2.552 | -3.860 | -1.326 |
| BJP76_RS18205 | glycosyltransferase family 2 protein | -2.421 | -3.759 | 0.064 |
| BJP76_RS18800 | hypothetical protein | -2.062 | -2.011 | 0.156 |
| BJP76_RS18905 | NnrU family protein | -2.903 | -3.952 | -0.672 |
| BJP76_RS20900 | SulP family inorganic anion transporter | -4.262 | -3.121 | 0.965 |
| BJP76_RS21715 | hypothetical protein | -2.515 | -2.166 | 0.889 |
| BJP76_RS21720 | hypothetical protein | -3.896 | -3.853 | -0.035 |
| BJP76_RS23510 | elongation factor G-like protein EF-G2 | -2.453 | -3.972 | -1.678 |
| BJP76_RS23640 | hypothetical protein | -2.354 | -2.373 | -0.844 |
| BJP76_RS23660 | cutinase family protein | -2.503 | -2.566 | -0.489 |
| BJP76_RS23665 | aldehyde dehydrogenase family protein | -2.883 | -2.442 | -0.257 |
